# Virus Disinfection from Environmental Water Sources Using Living Engineered Biofilm Materials

**DOI:** 10.1002/advs.201903558

**Published:** 2020-05-22

**Authors:** Jiahua Pu, Yi Liu, Jicong Zhang, Bolin An, Yingfeng Li, Xinyu Wang, Kang Din, Chong Qin, Ke Li, Mengkui Cui, Suying Liu, Yuanyuan Huang, Yanyi Wang, Yanan Lv, Jiaofang Huang, Zongqiang Cui, Suwen Zhao, Chao Zhong

**Affiliations:** ^1^ Materials and Physical Biology Division School of Physical Science and Technology ShanghaiTech University Shanghai 201210 China; ^2^ iHuman Institute ShanghaiTech University Shanghai 201210 China; ^3^ School of Life Science and Technology ShanghaiTech University Shanghai 201210 China; ^4^ State Key Laboratory of Virology Wuhan Institute of Virology Chinese Academy of Sciences Wuhan 430071 China; ^5^ Wuxi Biologics Co., Ltd. Suzhou 215100 China; ^6^ Shanghai Institute of Applied Physics Chinese Academy of Sciences Shanghai 201800 China; ^7^ Present address: Materials Synthetic Biology Center Shenzhen Institute of Synthetic Biology Shenzhen Institutes of Advanced Technology Chinese Academy of Sciences Shenzhen 518055 China; ^8^ Present address: CAS Key Laboratory of Quantitative Engineering Biology Shenzhen Institute of Synthetic Biology Shenzhen Institutes of Advanced Technology Chinese Academy of Sciences Shenzhen 518055 China

**Keywords:** engineered biofilms, living materials, water disinfection

## Abstract

Waterborne viruses frequently cause disease outbreaks and existing strategies to remove such viral pathogens often involve harsh or energy‐consuming water treatment processes. Here, a simple, efficient, and environmentally friendly approach is reported to achieve highly selective disinfection of specific viruses with living engineered biofilm materials. As a proof‐of‐concept, *Escherichia coli* biofilm matrix protein CsgA was initially genetically fused with the influenza‐virus‐binding peptide (C5). The resultant engineered living biofilms could correspondingly capture virus particles directly from aqueous solutions, disinfecting samples to a level below the limit‐of‐detection for a qPCR‐based detection assay. By exploiting the surface‐adherence properties of biofilms, it is further shown that polypropylene filler materials colonized by the CsgA‐C5 biofilms can be utilized to disinfect river water samples with influenza titers as high as 1 × 10^7^ PFU L^−1^. Additionally, a suicide gene circuit is designed and applied in the engineered strain that strictly limits the growth of bacterial, therefore providing a viable route to reduce potential risks confronted with the use of genetically modified organisms. The study thus illustrates that engineered biofilms can be harvested for the disinfection of pathogens from environmental water samples in a controlled manner and highlights the unique biology‐only properties of living substances for material applications.

## Graphical abstract

A simple, efficient, and environmentally friendly approach to achieve highly selective disinfection of specific viruses with living engineered biofilm materials from virus‐polluted water is reported. The engineered biofilms can disinfect influenza virus from contaminated water to a level below the limit‐of‐detection for a qPCR‐based detection assay, thus protecting mammalian cells against virus infections efficiently.

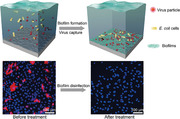

## 1 Introduction

Waterborne disease outbreaks from viral pathogens occur each year worldwide,^[^ ^1^ ^]^ and the disinfection of viral pathogens is recognized as a critical but challenging process in water treatment.^[^ ^2^, ^3^ ^]^ Conventional technologies to address this problem like chlorine and ozone treatment are chemically intensive and may produce dangerous disinfection byproducts,^[^ ^4^ ^]^ while use of UV light illumination and high‐pressure filtration are energy intensive and can fail against UV‐resistant viruses like adenovirus.^[^ ^5^ ^]^ Further, some viruses are resistant to the chemicals used in water disinfections,^[^ ^6^ ^]^ and the sizes of some viruses are too small to be filtered by conventional membranes.^[^ ^7^, ^8^ ^]^ Thus, the development of a new generation of simple, efficient and environmentally friendly virus disinfection strategies that are complementary to existing technologies would be highly demanded. To this end, water treatment experts have suggested the exciting possibility of future technologies that might achieve exquisite molecular‐level specificity for selective viral binding to materials functionalized with, for example, host receptor proteins of specific viruses.^[^ ^2^ ^]^

Biofilms—consortia of microbial cells embedded in a protective extracellular matrix^[^ ^9^ ^]^—have been used in water treatment for a long time.^[^ ^10^ ^]^ For example, naturally occurring biofilms are frequently harvested for the remediation of toxic compounds and heavy metals.^[^ ^10^ ^]^ Inspired by these historical applications of biofilms in water treatment, we here propose and explore the concept of engineering biofilms as living materials for virus disinfection based on the extracellular assembly of genetically engineered proteins in the biofilm matrix to enable specific interactions with and thus robust capture of targeted pathogenic viruses. Ideally, such an approach would achieve highly efficient and selective disinfection of targeted viruses with very low energy inputs and minimal infrastructure requirements. Moreover, this living biofilm platform would harness the unique properties of living systems, including genetic programmability, self‐regeneration, and evolutionary and environmental adaptability, attributes offering advantages over conventional water treatment technologies in terms of scalability for biomanufacture and deployment, for example, to prevent transmission of waterborne viral pathogens at geographically remote or otherwise inaccessible sites during epidemic outbreaks.

## 2 Results and Discussion

**Figure 1:**
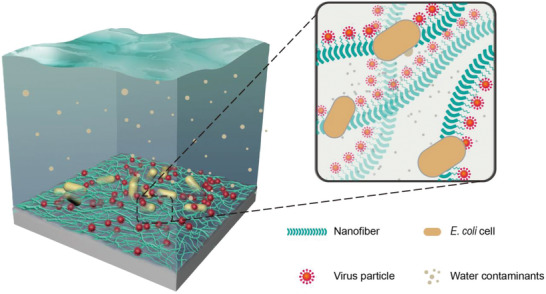
Schematic diagram of the engineered biofilms for disinfecting viruses from water. Genetically engineered *E. coli* biofilms specifically bind with and thus disinfect water transmission influenza virus from river water through functional extracellular amyloid nanofibers comprising CsgA‐C5 monomers. The C5 peptide, previously identified by phage display,^[^ ^21^ ^]^ was rationally fused with the CsgA protein to form CsgA‐C5 fusion monomer. CsgA‐C5 proteins can be secreted out of the bacteria cells and self‐assemble into the amyloid fibers comprising the extracellular matrix of engineered biofilms.

Our rational design for engineering *Escherichia coli* biofilms for disinfection of virus in water was based on CsgA proteins, a major component of *E. coli* biofilms.^[^ ^11^ ^]^ CsgA protein monomers are secreted from bacterial cells and can self‐assemble into amyloid nanofibers.^[^ ^12^ ^]^ Notably, genetically modified bacterial biofilms have recently found a wide range of interesting applications in catalysis, biosensor, and bioremediation as engineered living materials.^[^ ^13^, ^14^, ^15^, ^16^, ^17^, ^18^, ^19^, ^20^ ^]^ As an initial proof‐of‐concept for viral binding in this study, we choose the influenza virus (H1N1) as a model, and engineered fusion monomers that combined CsgA with a known influenza‐virus‐binding peptide—here denoted as C5—which was previously identified via phage display; C5 (amino acid sequence: ARLPR) has been shown to bind to hemagglutinin (HA), a membrane glycoprotein of influenza virus^[^ ^21^ ^]^ (**Figure** **1**).

**Figure 2:**
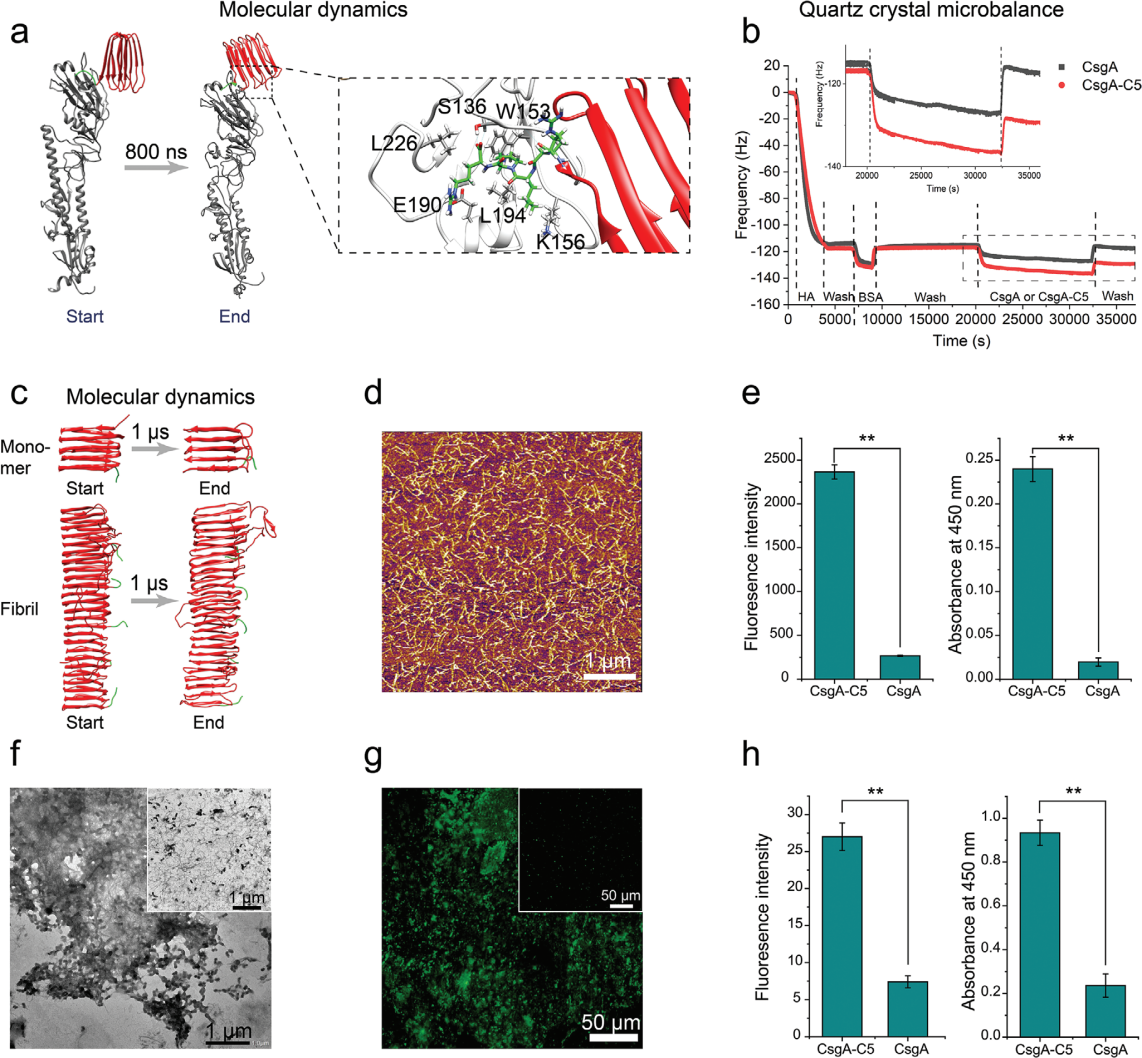
Binding between CsgA‐C5 nanofibers and influenza virus (H1N1). a) Start and end simulated structures representative of CsgA‐C5 monomer interacting with hemagglutinin (HA, the major membrane glycoprotein of influenza virus) revealed by molecular dynamics simulations. Simulation time was 800 ns. The zoomed‐in image shows the detailed interaction between CsgA‐C5 monomer and hemagglutinin. The interactions among the key residues include hydrogen bonding interactions (between R136 (CsgA‐C5) and S1 36 (HA) and between R136 (CsgA‐C5) and E190 (HA)) and hydrophobic interactions (between L134 (CsgA‐C5) and K156 (HA), among P135 (CsgA‐C5), W153(HA), and L194 (HA), and between R136 (CsgA‐C5) and L226 (HA)). b) QCM analysis of the affinity strength between CsgA‐C5 monomers and hemagglutinin. The inset image shows the zoomed‐in curves in the range of 18 000–36 000 s. c) The start and end simulated structures for the CsgA‐C5 monomer (top) and fibril (bottom) revealed by molecular dynamics simulations. Simulation time was 1 µs. d) AFM height image showing the morphology of self‐assembled CsgA‐C5 fibers. e) Confocal immunofluorescence intensity (left) and ELISA (right) analysis to quantitatively assess the binding of CsgA‐C5 and CsgA nanofibers to hemagglutinin. Results are means ± s.e.m. of three independent samples (*n* = 3). ^**^ *P* < 0.01, Student's *t*‐test. f) TEM and g) immunofluorescence images of the CsgA‐C5 and CsgA (inset) fibers binding with whole viruses. h) Immunofluorescence intensity (left) and ELISA (right) analysis to assess the binding of CsgA‐C5 (and CsgA) nanofibers with whole virus particles. Results are means ± s.e.m. of three independent samples (*n* = 3). ^**^ *P* < 0.01, Student's *t*‐test.

We initially used computational approaches to assess the reactivity of CsgA‐C5 fusion monomers. Although previous work has shown that the C5 influenza‐virus‐binding peptide has a high affinity to hemagglutinin, we needed to confirm that C5 could still interact with hemagglutinin after being fused with the CsgA protein. To such ends, we first used MODELLER^[^ ^22^, ^23^ ^]^ to build the homology models of CsgA‐C5 and Glide^[^ ^24^ ^]^ to get the complex of the monomer CsgA‐C5 and hemagglutinin (PDB ID: 1HGG). Molecular dynamics simulations of the interaction between a CsgA‐C5 fusion monomer and hemagglutinin by GROMACS^[^ ^25^ ^]^ indicated that these two proteins interact strongly: the bound complex structure was stable even after 800 ns (**Figure** **2**a); the binding energy was calculated using the molecular mechanics/generalized born surface area (MM/GBSA) method,^[^ ^26^ ^]^ and the Δ*G*_bind_ value was about −62 ± 22 kcal mol^−1^, which is similar to the binding energy between biotin analogous and avidin.^[^ ^27^ ^]^

We used *E. coli* to recombinantly express CsgA monomers and CsgA‐C5 monomers, and following cell lysis, these proteins were purified following standard protocols^[^ ^23^ ^]^ and migrated as single bands at 14.1 and 14.6 kDa, respectively, under SDS‐polyacrylamide gel electrophoresis (SDS‐PAGE) and western blotting (Figure S1, Supporting Information). We then conducted quartz crystal microbalance (QCM) experiments wherein fresh eluted CsgA and fusion CsgA‐C5 monomers were exposed to silicon substrates that were coated with hemagglutinin. Compared with CsgA control monomers, CsgA‐C5 monomers showed substantially enhanced absorption: the mass of CsgA‐C5 monomers absorbed on the HA‐coated substrate was about four times as much as that of the absorbed CsgA monomers (Figure 2b). This result indicates that the C5 peptide is essential for the interaction between CsgA and hemagglutinin, and confirms that CsgA‐C5 fusion monomers retain the hemagglutinin‐binding activity of the C5 peptide.

We next investigated whether the presence of the C5 peptide might affect the overall structure of CsgA amyloid cores. We again initially built molecular dynamics models: one representing the monomeric and one representing the fibrillar states of the CsgA‐C5 structures (Figure 2c). Simulations of the monomeric proteins (1 µs) and the fibrillar states (1 µs) indicated that the core amyloid structure comprising the CsgA‐C5 fusion monomers does not substantially diverge from that of a typical CsgA amyloid structure. The models also suggested that CsgA‐C5 monomers should assemble into stable amyloid structures dominated by the CsgA domains, with the C5 peptides displayed external to the amyloid core. Collectively, these results thus validate the rationality of our design—the influenza virus‐binding sites are fully exposed, which should allow binding of influenza hemagglutinin with the C5 peptide of the fibrillar amyloids.

To experimentally validate the results from our simulations, we next tested if the CsgA‐C5 fusion monomer proteins could assemble into fibers. ThT (thioflavin T) (Figure S2, Supporting Information) and Congo red assays (Figure S3, Supporting Information) showed that CsgA‐C5 and CsgA proteins exhibited amyloid features. Further, both the CsgA‐C5 and CsgA monomers were able to self‐assemble into long and stable fibers, as confirmed by transmission electron microscopy (TEM) and atomic force microscopy (AFM) (Figure 2d; Figure S4, Supporting Information). In addition, X‐ray fiber diffraction data (Figure S5, Supporting Information) revealed the typical cross‐beta diffraction patterns characteristic of amyloid structures^[^ ^28^ ^]^ for both the CsgA‐C5 and CsgA amyloid nanofibers.

We next used immunofluorescence and enzyme‐linked immunosorbent assay (ELISA) to test if the self‐assembled amyloid nanofibers retained their capacity for binding hemagglutinin. We first quantified the biomass densities of the CsgA and CsgA‐C5 nanofiber samples formed on the substrates by measuring the initial and remaining concentrations of protein solutions before and after nanofiber coating formation on the substrates. Note that the biomass densities of the CsgA and CsgA‐C5 nanofiber coatings were identical (around 0.1 mg cm^−2^) (Table S1, Supporting Information), however, the affinity of CsgA‐C5 fibers for hemagglutinin was increased by tenfold compared to CsgA fibers (Figure 2e), in consistence with the aforementioned result based on QCM. We also investigated if CsgA‐C5 fibers could bind with intact virus particles. TEM images demonstrated that apparently all of the CsgA‐C5 protein fibers could specifically bind with influenza virus particles; this was in sharp contrast to the CsgA controls, which absorbed very few virus particles (Figure 2f). Immunofluorescence microscopy images also showed a similar result (Figure 2g): the glass substrate coated with CsgA‐C5 fibers was covered with viruses, whereas very few viruses were absorbed on the CsgA‐coated glass. Quantification using ELISA and immunofluorescence intensity analysis (Figure 2h) showed that the affinity of CsgA‐C5 nanofibers for hemagglutinin was about fourfold greater than that of the CsgA fibers.

**Figure 3:**
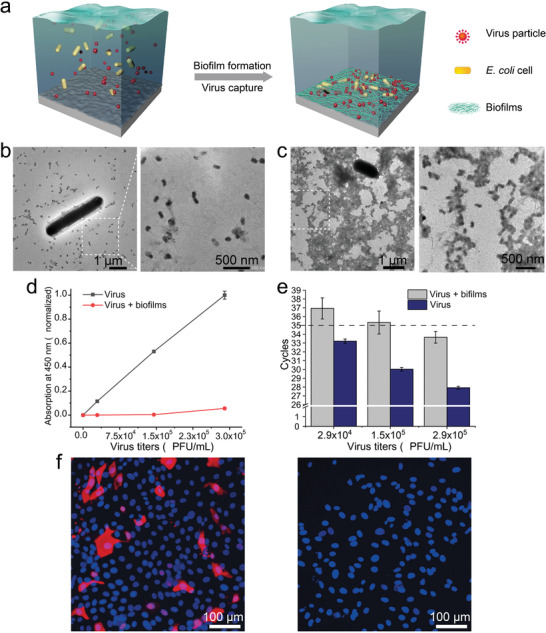
Virus disinfection using engineered functional biofilms. a) Schematic of engineered *E. coli* cells that self‐assemble into biofilms upon expression and extracellular secretion of CsgA‐C5 proteins and capture viruses present in water with extracellular nanofibers. b) TEM images of the unbound status of bacterial cells and viruses (before biofilm formation) (an image of the zoomed‐in area is shown at the right). c) TEM images of the CsgA‐C5 biofilms binding with virus particles (an image of the zoomed‐in area is shown at the right). d) ELISA and e) qPCR analysis of supernatants from a gradient series of virus titers samples that were incubated with biofilms. Results show means ± s.e.m. of three independent samples (*n* = 3). f) Biofilms were exposed to influenza virus samples (7 × 10^4^ PFU mL^−1^) in PBS, and the sample supernatant was then used to inoculate cells from the influenza‐susceptible MDCK (Madin–Darby canine kidney) cell line. Inoculated cells were then analyzed using a mouse monoclonal antibody against the influenza virus nucleoprotein to detect virus particles that had successfully infected cells.

Having established at the protein level that our CsgA‐C5 amyloid fibers form strong interactions with influenza virus particles, we next explored the use of engineered *E. coli* biofilms with the CsgA‐C5 fibers to capture viruses directly from aqueous solutions (**Figure** **3**a). The virus particles were added to the culture media and then co‐cultured with the engineered *E. coli* cells. Prior to induction of biofilm formation, the virus particles were freely distributed around bacteria cells (Figure 3b). After induction for 72 h, we found that the CsgA‐C5 fibers of the adhered *E. coli* biofilms bound with many influenza virus particles (Figure 3c). In contrast, very few virus particles were captured by control CsgA fibers in biofilms (Figures S6 and S7, Supporting Information).

We next explored the influenza‐virus‐binding capacity of the engineered biofilms by exposing them to a series of influenza virus titers (ranging from 2.9 × 10^4^ to 2.9 × 10^5^ PFU mL^−1^). We collected the supernatants from the samples, and AFM analysis showed that there were many virus particles in the control supernatants (from CsgA biofilm samples) but hardly any in the supernatant from the CsgA‐C5 biofilm samples, even at very high viral titers (Figure S8, Supporting Information). Further, ELISA detected a clear concentration‐dependent increase in viral signal for the control samples that were not exposed to biofilms (3 days at 29 °C), while only a very low signal was detected for the supernatants from the CsgA‐C5 biofilm samples, with a slight increase evident for only the highest titer sample (Figure 3d). We also analyzed these samples with the sensitive qPCR assay (Figure S9, Supporting Information) and noted the same trend: only the highest viral titer biofilm‐incubated sample had a signal above the detection limit for the commercial Venzyme Cham QTM SYBR Color qPCR Master Mix kit that we applied for this analysis (Figure 3e).

Notably, both the ELISA and qPCR analyses revealed a small increase in the viral signal for the highest viral titer sample, suggesting possible saturation of the viral‐particle‐binding capacity of the C5 peptides present in these biofilms. Although it is difficult to precisely control the spatial distribution of C5 monomers in these living biofilms, it should in theory be possible to combine different bacterial seeding rates with similar viral concentration series to more precisely ascertain the saturation levels of these materials. Importantly, considering the potential water‐pathogen‐disinfectant applications, we also tested whether mammalian cells could still become infected after virus‐infected water was treated with the engineered CsgA‐C5 biofilms. Specifically, we exposed the highly influenza‐susceptible Madin–Darby canine kidney (MDCK) cells to control or post‐biofilm incubation supernatant from the 7 × 10^4^ PFU mL^−1^ viral titer samples, and immunofluorescence analysis with an antibody against influenza virus nucleoprotein indicated that no cells became infected with the post‐biofilm incubation supernatant. In sharp contrast, many of the cells exposed to the control supernatant had strong signals indicating virus infection (Figure 3f). A similar result was also confirmed by hemagglutination inhibition assay in which the sediment of chicken red blood cells to the well bottom, a sign indicating the absence of viral HA proteins in the solution, was found for both of the biofilm‐treated and virus‐free negative control solutions (Figure S10, Supporting Information). Taken together, these results revealed that our engineered biofilms could bind and thus efficiently eliminate influenza viral particles from aqueous solutions to an extent that apparently precludes infection of highly susceptible mammalian cells.

**Figure 4:**
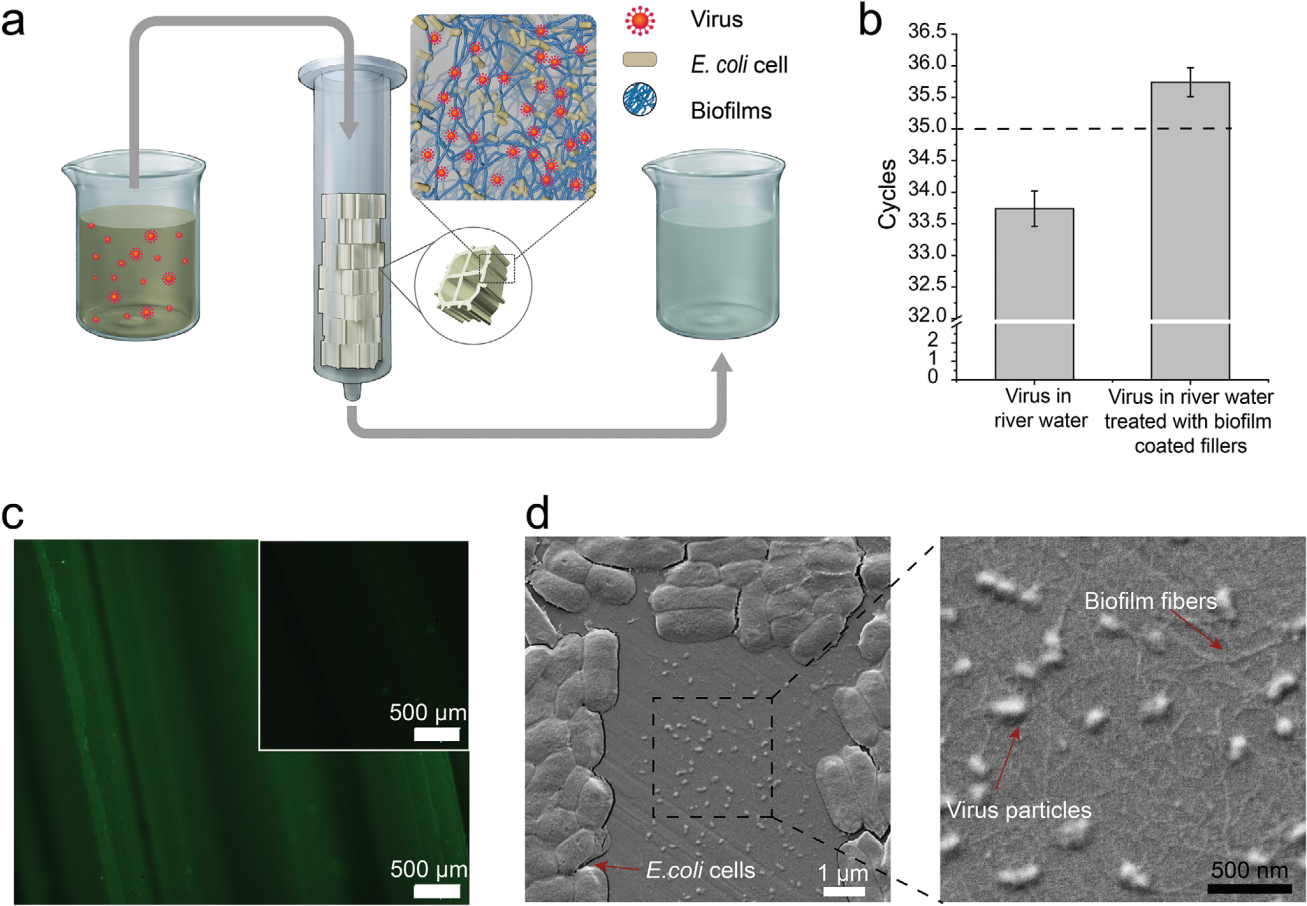
Integrating engineered functional biofilms with industrial filler materials for virus elimination from river water. a) Schematic for polypropylene industrial filler material colonized by our engineered CsgA‐C5 biofilms and used to eliminate viruses from river water. b) qPCR analysis of field samples after virus‐spiked river water samples were passed over the immobilized biofilms. Results show means ± s.e.m. of three independent samples (*n* = 3). c) Immunofluorescence images of the biofilm‐coated polypropylene industrial filler material after passage of the field water samples, stained against hemagglutinin. The inset image refers to the bare filler materials as a control test sample. d) SEM images of the virus particles bound to the CsgA‐C5 biofilms (zoomed‐in images are shown at the right). *E. coli* cells, amyloid fibers, and virus particles are indicated with arrows.

By exploiting the fact that *E. coli* biofilms can inherently adhere to diverse substances and complex structures,^[^ ^19^, ^29^ ^]^ we next grew CsgA‐C5 biofilms on the polypropylene surfaces of intricate gear‐like industrial filler material and tested their capacity to capture virus particles from river water (**Figure** **4**a). Congo red staining confirmed that the CsgA‐C5 biofilms could successfully grow on the surfaces (Figure S11, Supporting Information). Further, using a previously reported Congo red binding assay,^[^ ^30^ ^]^ we determined that about 1.86 mg CsgA‐C5 biofilms formed on one individual filler (Figure S12, Supporting Information). We next spiked river water samples with an influenza virus titer of 1 × 10^7^ PFU L^−1^, a level much higher than reported human virus titers in river water (which range from 10^2^ to 10^5^ PFU L^−1^).^[^ ^31^, ^32^ ^]^ We then passed the water samples over the filler materials, and qPCR analysis showed that the virus could be easily detected in the unfiltered control samples (virus‐spiked river water) but was undetectable in the filtrate sample (Figure 4b). Further, both immunofluorescence (Figure 4c) and scanning electron microscopy (SEM) (Figure 4d) images demonstrated that the virus particles from the river water samples had been attached to the nanofibers of the filler‐immobilized CsgA‐C5 biofilms.

To determine the maximum capacity of the filtration unit, we incubated five pieces of the CsgA‐C5 biofilm‐coated industrial fillers (diameter = 11 mm) in the presence of 10 mL excessive amount of influenza virus particles in river water (1.84 × 10^5^ PFU mL^−1^) at room temperature for at least 2 h. After sufficient binding between CsgA‐C5 biofilms and virus particles, the solution in the column was allowed to flow out at an average rate of 0.67 mL s^−1^. The titers of virus solution were next determined by qPCR assay, and the results showed that the titers changed from 1.84 × 10^5^ to 7.3 × 10^4^ PFU mL^−1^ after the virus solution was treated with CsgA‐C5 biofilm‐coated fillers. The total capacity of the fillers could be calculated with a value of 1.11 × 10^6^ PFU. Therefore, the maximum capacity of the filtration unit could be determined with an average value of 2.22 × 10^5^ PFU per filler. We next used five similar pieces of biofilm‐coated fillers to treat virus solution at a titer of 1.0 × 10^4^ PFU mL^−1^ for assessing the amount of samples that one filtration unit process before its virus capture capability drops. Our results indicated that this system could at most treat a total of 50 mL influenza virus‐polluted water (10 mL per filler): when the volume of virus solution applied increased above 50 mL, the capacity of virus disinfection from river water using the biofilm‐coated fillers dropped, as revealed by qPCR assay (cycles < 35) (Figure S13, Supporting Information).

After confirming the feasibility of water disinfection using living engineered biofilms, we turned to assess the potential biosafety concerns brought about with the use of genetically modified organisms (GMOs). To such end, we engineered a quorum sensing‐enabled suicide gene circuit into our engineered bacteria.^[^ ^33^ ^]^ Specifically, a suicide gene circuit consisting of a lysis protein (*ϕ*x174E), activated by the expression of LuxI‐LuxR, was introduced into our engineered *E. coli*. The *luxI* gene under a J23108 constitutive promoter, producing an acylhomoserine lactone (a quorum sensing signal), can activate the suicide gene expression regulated by LuxR repressor. In this way, when the cell density reaches a critical threshold, expression of the toxin protein (*ϕ*x174E) will be initiated, thus resulting in bacterial death. As is shown in figure S14 in the Supporting Information, the growth curve of the engineered bacteria containing *ϕ*x174E clearly showed an autonomous inhibition effect when cell density (OD_600_) reached 0.35, while the growth of the control bacteria (without a suicide gene circuit) showed a continuous growth mode without any inhibition effect (Figure S14, Supporting Information). To test whether introduction of the suicide gene circuit would affect the performance of virus capture, we incubated the engineered bacteria containing the suicide gene with virus particles (3.5 × 10^7^ PFU L^−1^) and found that the engineered bacteria could still efficiently express biofilms and effectively eliminate the virus particles in the supernatant (Figure S15, Supporting Information). Collectively, these results thus showed the feasibility of introducing a suicide gene circuit for controlling the fate of engineered bacterial, thus illustrating a viable route to address the biosafety concerns raised by the use of genetically modified organisms including engineered bacteria.

We here used biofilms programmed with a virus‐protein‐binding peptide to endow engineered biofilms the ability to efficiently capture viruses from river water. Compared with conventional water treatment methods, our strategy is green, low costs, and low energy inputs. Essentially, our engineered CsgA‐C5 biofilms achieved strong and specific disinfection of the target virus from water samples to a level that precluded infection of cells known to be highly susceptible to influenza infection. Extending the concept to showcase the biology‐specific functional properties of a genuinely living material, we used our functional biofilms to grow on and colonize polypropylene inserts in a way that also robustly disinfected viruses from river water samples. Our initial proof‐of‐concept demonstrations targeted the influenza virus as a model, but clearly any virus‐binding peptide or protein‐based moiety (e.g., host receptor proteins, antibodies) should be suitable for fusion with CsgA proteins to enable similar biofilm‐mediated disinfection for other viruses. To reduce potential risks confronted with the use of genetically modified organisms, we have applied a suicide gene circuit in our engineered strain that can successfully restrict the growth of bacterial in a controlled manner. Beyond strictly controlling bacterial growth, other gene circuits such as “deadman” and “passcode” could also be possibly engineered into the bacteria to control cell viability.^[^ ^34^ ^]^ Alternatively, another possible approach is to genetically delete the cell‐wall synthesis gene in the engineered strain (for example, the dapA gene), rendering the growth of the engineered bacterial dependent on exogenous diaminopimelate (DAP).^[^ ^35^ ^]^ In this way, any escaped engineered cells would not survive in the environment without providing sufficient DAP, as DAP in the surroundings is insufficient to support the growth of auxotrophs.^[^ ^36^ ^]^

Given that bacterial biofilms can be genetically modified and considering the modularity of fusion amyloid monomers, it should be relatively straightforward to deploy other CsgA fusion proteins which combine the amyloid nanofiber self‐assembly and biofilm‐forming capacities of CsgA domains with additional functional domains that selectively bind to (and thus sequester) other viruses and perhaps even other classes of waterborne pathogens (e.g., binding to surface or other exposed proteins of bacteria like *Vibrio cholera* ^[^ ^37^ ^]^ and protozoans like *Giardia lamblia* ^[^ ^38^ ^]^). It should also be possible to further engineer the material performance of the biofilms themselves by fusing the CsgA‐pathogen binding monomers to other protein domains that can alter biofilm mechanical properties tailored for specific applications. Looking beyond *E. coli* and in light of our previously reported work demonstrating that the FDA generally‐regarded‐as‐safe bacterium *Bacillus subtilis* and its TasA amyloid proteins can be engineered and generally functionalized as a biofilm living material platform,^[^ ^17^ ^]^ we anticipate that this generally pathogen‐binding biofilm concept could even find applications in other application domains, for example, applying engineered biofilms in the gut to capture and digest toxic gut pathogens or viruses.

## 3 Conclusion

Our living materials are complementary to existing conventional technologies used for water disinfection, and an important point of contrast of our living materials versus conventional technologies like hazardous chemical treatment or ultrafiltration relates to technological deployment. Given that biofilm disinfection materials can be grown as needed in situ, they may be easier to distribute to remote areas (where various target‐pathogen‐functionalized biofilms could be stored as culture sample libraries), especially in difficult‐to‐access areas during epidemic outbreaks. That is, rather than requiring the transport of dangerous chemicals, energy‐intensive filtration equipment, strong generators, and trained personal to properly and safely implement and manage pathogen‐disinfection water treatment processes, local inhabitants of such areas could for example grow living engineered biofilms in their own buckets and other water vessels.

## Conflict of Interest

The authors declare no conflict of interest.

## Author Contributions

J.P. and Y.L. contributed equally to this work. C.Z. directed the project. J.P. conceived the technical details and designed the experiments. J.P. carried out the experiments including plasmid construction, protein purification, TEM, AFM, ELISA, immunofluorescence, biofilm culturing, and data analysis. Y.L. performed the experiments including RNA extraction, qPCR, cell culture and virus infection, immunofluorescence, molecular dynamics, and data analysis. S.Z. and K.D. helped the molecular dynamics and related computational biology analysis. Z.C. and C.Q. helped the experiment part of cell culture and virus infection, B.A. constructed the plasmids. J.P., Y.L., and C.Z. analyzed the data, discussed results, and wrote the manuscript with help from all the authors. All the authors revised the manuscript.

## Supporting information

Supporting Information
