# Supplementary material for: Virus Disinfection from Environmental Water Sources Using Living Engineered Biofilm Materials
Source: Adv Sci (Weinh). 2020 May 22;7(14):1903558. doi: 10.1002/advs.201903558 (PMC7375245; doi:10.1002/advs.201903558)
Supplement: Supplementary file 1 — Supporting Information [file ADVS-7-1903558-s001.pdf]

Supplementary Information for:

Virus Disinfection from Environmental Water Sources Using Living Engineered Biofilm Materials

Jiahua Pu[1†], Yi Liu[1†], Bolin An[1], Yingfeng Li[1,2], Xinyu Wang[1], Jicong Zhang[1], Kang Din[3,5], Chong Qin[4,7], Ke Li[1], Mengkui Cui[1,2], Suying Liu[1,2,6], Yuanyuan Huang[1], Yanyi Wang[1], Yanan Lv[2,5,6], Jiaofang Huang[1], Zongqiang Cui[4], Suwen Zhao[3,5], Chao Zhong[1,8*]

[1] Materials and Physical Biology Division, School of Physical Science and Technology, ShanghaiTech University, Shanghai 201210, China.

[2] University of Chinese Academy of Sciences, Beijing 100049, China.

[3] iHuman Institute, ShanghaiTech University, Shanghai 201210, China.

[4] State Key Laboratory of Virology, Wuhan Institute of Virology, Chinese Academy of Sciences, Wuhan 430071, China.

[5] School of Life Science and Technology, ShanghaiTech University, Shanghai 201210, China.

[6] Shanghai Institute of Applied Physics, Chinese Academy of Sciences, Shanghai 201800, China.

[7] Wuxi Biologics Co., Ltd., Suzhou 215100, China

[8] Present address: Materials Synthetic Biology Center, Shenzhen Institute of Synthetic Biology, Shenzhen Institutes of Advanced Technology, Chinese Academy of Sciences, Shenzhen 518055, China

[†] These authors contributed equally to this work.

[*]Corresponding author. Email: zhongchao@shanghaitech.edu.cn

Materials and Methods:

Table of Contents

1. Plasmids
2. Strains
3. Protein expression and purification
4. Thioflavin T (ThT) assay
5. Quartz Crystal Microbalance (QCM)
6. Congo red staining
7. X-ray fiber diffraction
8. Biofilms cultivation
9. Cell density detection and biofilms quantification
10. Transmission electron microscopy (TEM) imaging
11. Scanning electron microscopy (SEM) imaging
12. Atomic force microscopy (AFM) imaging
13. Biomass quantification of the CsgA or CsgA-C5 nanofiber coatings formed on glass substrates
14. Enzyme linked immunosorbent assay (ELISA)
15. Cell culture and virus infection
16. Immunofluorescence imaging
17. Molecular dynamics simulation
18. Homologue modelling
19. RNA extraction, reverse transcription, and qPCR
20. Biomass quantification of CsgA-C5 biofilms formed on industrial fillers.
21.Virus disinfection from river water using CsgA-C5 biofilm-coated industrial fillers.

Supplementary Figure 1: SDS-PAGE and western blot of the purified CsgA and CsgA-C5 proteins

Supplementary Figure 2: ThT assay

Supplementary Figure 3: Congo red staining of CsgA and CsgA-C5 amyloid fibers

Supplementary Figure 4: AFM and TEM images of CsgA and CsgA-C5 amyloid fibers

Supplementary Figure 5: XRD of CsgA and CsgA-C5 amyloid fibers

Supplementary Figure 6: TEM images of CsgA biofilms binding influenza virus particles.

Supplementary Figure 7: Immunofluorescence images of the CsgA-C5 biofilms and CsgA biofilms binding influenza virus particles.

Supplementary Figure 8: AFM images of the supernatant of influenza virus solutions at gradient titers exposed to CsgA-C5 biofilms.

Supplementary Figure 9: The qPCR amplification curve of influenza viruses at different titers.

Supplementary Figure 10: Hemagglutination inhibition assay of influenza viruses before and after biofilm treatment.

Supplementary Figure 11: Congo red staining of the industrial filler coated with CsgA-C5 biofilms.

Supplementary Figure 12: Linear curve of the amount of Congo red absorbed by CsgA-C5 biofilms versus the mass of the biofilms.

Supplementary Figure 13: qPCR analysis of the virus-spiked river water treated with CsgA-C5 biofilm coated fillers

Supplementary Figure 14: Growth curve of the bacteria with (black) or without (red) suicide gene circuit.

Supplementary Figure 15: Virus disinfection from river water using engineered bacteria introduced with suicide gene circuit.

Supplementary Figure 16. Plasmid maps of the constructed plasmids used to control the growth of the genetically engineered bacteria.

Supplementary Table 1: Density determination of protein coatings formed on glass substrates.

Supplementary Table 2: Synthetic materials used in this work.

Supplementary Table 3: Plasmids used in this work.

Supplementary Table 4: Strains used in this work.

Supplementary Table 5: Primers used to detect influenza viruses in solution via qPCR in this work.

Plasmids:

(1) Construction of tetracycline (Tc)-inducible plasmid (pZA-CmR-rr12-pL(tetO)-ssCsgA-C5-CsgC-CsgG)

pZA-CmR-rr12-pL(tetO)-ss-CsgA-C5-CsgC-CsgG plasmid was constructed based on the pZA-CmR-rr12-pL(tetO)-CsgA plasmid obtained from the Lu lab at MIT[1]. Briefly, a recombinant gene combining CsgA and its biological secretion signal (ss) sequence appended with C-terminal C5 peptide tags was obtained by polymerase chain reaction (PCR). CsgC and CsgG fragments were amplified from MG1655 genome by PCR reaction. The pZA-CmR-rr12-pL(tetO)-CsgA plasmid was digested with restriction endonucleases KpnI/MluI, and then the ss-CsgA-C5, CsgC and CsgG fragments we inserted into the linearized pZA-CmR-rr12-pL(tetO) vector via Gibson Assembly (New England Biolabs). Plasmid sequence was confirmed by restriction digest and sequenced by Sangon Biotech (Shanghai).

(2) Construction of IPTG-inducible plasmid (pET-22b-CsgA-C5)

Recombinant gene combining CsgA and C5 peptide was obtained by polymerase chain reaction (PCR). The commercially available plasmid pET-22b was purchased from Novagen and digested with restriction endonucleases NdeI/XhoI. The CsgA-C5 fragment was then inserted into peT-22b expression vector via T4 ligase (New England Biolabs). Plasmid sequence was confirmed by restriction digest and sequenced by Sangon Biotech (Shanghai).

(3) Construction of IPTG-inducible plasmid (pET-22b-CsgA)

A recombinant gene combining CsgA and polyhistidine tags was obtained by polymerase chain reaction (PCR). The commercially available plasmid pET-22b was purchased from Novagen and digested with restriction endonucleases NdeI/XhoI, and then the CsgA fragment was inserted into pET-22b expression vector via T4 ligase (New England Biolabs). Plasmid sequence was confirmed by restriction digest and sequenced by Genewiz (Suzhou).

The synthetic materials used in this work that constitute the plasmids are described in Supplementary Table S1. The plasmids are described in Supplementary Table S2. The strains are described in Supplementary Table S3. The primers used for qPCR are described in Supplementary Table S4.

Strains:

E. coli MG1655 PRO ΔCsgA ompR234 was obtained from Timothy K. Lu's lab, MIT. The detailed information for construction of this strain was described in a previous publication from the same group[1]. *E. coli* MG1655 PRO ΔCsgA generated by removing the kanamycin resistance cassette was described previously[2] (in order to free this antibiotic selection marker for subsequent usage). The strain was verified via PCR amplicon size using check primers for each locus. The ompR234 mutation was verified via sequencing of both strands of the amplicon before use. To obtain the CsgA-C5 biofilm forming cells, the pZA-CmR-rr12-pL(tetO)-ssCsgA-C5-CsgC-CsgG plasmid was transformed into *E. coli* MG1655 PRO ΔCsgA ompR234. To obtain the CsgA-C5 or CsgA protein over-expressing cells, pET-22b-CsgA-C5 or pET-22b-CsgA was transformed into commercially available BL21 (DE3) cells.

Protein expression and purification:

The recombinant plasmids containing pET-22b-CsgA-C5 and pET-22b-CsgA genes were transformed in BL21 (DE3) *E. coli* individually. Seed cultures (CsgA-C5 biofilms forming cells) were inoculated from frozen glycerol stocks and grown in LB medium using carbenicillin antibiotics at 50 μg/mL. Seed cultures were grown for 12 h at 37°C in 14-mL culture tubes (Falcon), with shaking at 220 rpm. Ten mL of the seed cultures were then inoculated in 1 L Luria-Bertani (LB) broth containing 50 μg/mL carbenicillin and were shaken at 220 rpm at 37°C for about 3 hours till the OD600 reached 0.8. Protein expression was induced with 1 mM IPTG for 1 hour. Cells were collected by centrifugation at 5,000 × *g* for 10 min, and every 5 g of the collected cell pellets were resuspended and lysed with 50 mL lysis buffer (8 M

guanidine hydrochloride (GdnHCl), 150 mM NaCl, 20 mM Tris·HCl, pH 7) at room temperature overnight. The insoluble portions of the lysates were removed through centrifugation at 20,000 × *g* for 45 min, and the separated supernatants were incubated with 1 mL Ni-NTA resin (Clontech) for 1 h at room temperature. Beads that bound with CsgA-C5 or CsgA were collected by centrifugation at 300 × *g* for 5 min, and washed with 50 mL of Tris·HCl buffer (20 mM Tris·HCl, 150 mM NaCl, pH=7.2). The mixed solutions were then loaded into 12 mL affinity columns (Sangon Biotech). GdnHCl was further washed away by adding 20 mL of excess Tris·HCl buffer (20 mM Tris·HCl, 150 mM NaCl, pH=7.2). Then, 100 mL washing buffer (20 mM Tris·HCl, 150 mM NaCl, 20 mM imidazole, pH=7.2) were passed through the column to remove contaminated proteins with 5 consecutive washing steps. Finally, 1-2 mL elution buffer (20 mM Tris·HCl, 150 mM NaCl, 1 M imidazole, pH = 7.2) was added to the column to elute target proteins. The protein concentration was detected via a Nanodrop 2000 Spectrophotometer (Thermo). The proteins were further confirmed via SDS-PAGE and western blot, following the protocols described in a previous work[3].

Thioflavin T (ThT) assay

A total of 200 μL of freshly purified protein solution containing protein monomers (1 mg/mL) were added into 96-well Flat Clear Bottom Black Polystyrene TC-treated Microplates (Corning, 3603). ThT buffer was then added at a final concentration of 20 μM. The signals were measured every 5 min after shaking for 5 s in a BioTek Synergy H1 Microplate Reader using BioTek GEN5 software set at 438 nm excitation and 495 nm emission with a 475 nm cutoff. The ThT fluorescence was normalized by $(F_i-F_0)/(F_{max}-F_0)$, where F_i stands for ThT intensity (fluorescence arbitrary unit) of samples, F_0 is the ThT background intensity, and F_{max} is the maximum ThT intensity of samples over the experiments.

Quartz Crystal Microbalance (QCM)

QCM measurements were performed with a Q-Sense Omega Auto system (Biolin Scientific). Silicon-coated quartz sensors were first sonicated in 2% (w/w) SDS

solution for 20 min and then rinsed with ultrapure water and ethanol. The crystals were dried under an N_2 stream, treated with UV/ozone for 30 min and mounted into the QCM chamber. Samples were introduced into the modules using a four-channel Ismatec ICP-N4 peristaltic pump. In QCM, changes in resonance frequency (ΔF) of a quartz crystal are recorded to measure the amount of the proteins deposited on the substrates. The crystal is excited at its fundamental frequency, approximately 5 MHz, and changes can be observed at the fundamental (n = 1), as well as overtone frequencies (n = 3, 5, 7, 9, and 11).

The hemagglutinin protein (5 mg/mL), provided by Shanghai Institute Biological Products Co. Ltd., was loaded onto the silicon-coated substrate at a rate of 2 μL/min followed by washing with TBS buffer (20 mM Tris·HCl, 150 mM NaCl, pH=7.4) at a rate of 10 μL/min, then 2 mg/mL BSA protein solution was loaded at a rate of 2 μL/min to block the substrate and 0.6 mg/mL CsgA-C5 or CsgA protein monomers were loaded onto the substrate at a rate of 2 μL/min followed by washing with TBS at a rate of 10 μL/min. Experiments were performed at 25°C.

Congo red staining

A total of 100 μL of 1 mg/mL CsgA-C5 or CsgA protein solutions sitting for a few days were spotted onto Protran BA83 nitrocellulose membranes (Whatman) with a dot blot manifold (Schleicher & Schuell Minifold-I Dot-Blot System). The membranes were then incubated with 20 mL of 0.002 (m/v %) Congo red solution at room temperature for 1 h. They were then washed three times with excess deionized water. The target proteins were confirmed using SDS-PAGE and western blot; data are shown in Supplementary Fig. 1a.

X-ray fiber diffraction

Mature fibrils formed by different protein samples were pelleted by centrifugation, followed by washing with excess distilled water several times to remove remaining salts. Fibril pellets were then suspended with 5 μL distilled water. Suspensions (2 μL) were pipetted between two fire-polished glass rods followed by drying for a couple of hours. The diffraction data were collected by an in-house X-ray machine equipped

with a Rigaku Micromax-007 X-ray generator and an R-Axis IV++ area detector.

Biofilms cultivation:

Seed cultures ($Tc_{Receiver}$/CsgA-C5) were inoculated from frozen glycerol stocks and grown in LB medium using chloramphenicol antibiotics at 34 μg/mL. Seed cultures were grown for 12 h at 37°C in 14-mL culture tubes (Falcon), with shaking at 220 rpm. Cells collected from the above seed cultures through centrifugation were resuspended with ddH_2O, and were then added into M63 medium at a volume ratio of 1:100, supplemented with 1 mM $MgSO_4$, 0.2% w/v glucose (hereafter referred to as glucose-supplemented M63). Chloramphenicol and tetracycline (Tc) were then added to the mixture at final concentrations of 34 μg/mL and 250 ng/mL, respectively. All liquid experimental cultures for biofilm growth were placed in an incubator (Shanghai Yiheng) and cultivated at 29°C without shaking.

To demonstrate that the CsgA-C5 biofilms can capture virus in solution as shown in Figure 3, gradient titers of virus were added into the culture solution directly and co-cultured for 3 days at 29°C before characterization.

Cell density detection and biofilms quantification

After cultivation of biofilms for 3 days at 29°C, the supernatant was removed, and the biofilms were scraped from the bottom of the culture dish and resuspended in 1 mL Tris·HCl buffer (pH=7.4). A total of 200 μL of the resuspended solution was taken out for OD600 cell number normalization. Then, 100 μL of 4 mg/mL Congo red was mixed with the remaining 900 μL bacteria solution or 900 μL ddH_2O to a final concentration of 0.4 mg/mL and incubated for 10 min at room temperature. Cells and curli with bound Congo red were spun down by centrifugation at 10,000 × *g* for 5 min in an Eppendorf Centrifuge 4424. Concentration of Congo red in supernatant was quantified by absorbance at 496 nm. The amount of Congo red bound by cells and curli were quantified by subtracting the $A_{496\ nm}$ of supernatant from $A_{496\ nm}$ of 0.4 mg/ml Congo red.

Transmission electron microscopy (TEM) imaging

For transmission electron microscopy (TEM), a 10 μL droplet of biofilm sample was directly deposited on a TEM grid (Zhongjingkeyi Technology, EM Sciences) for 4 min. The excess solution was wicked away with pieces of filter paper and the samples were rinsed twice with ddH_2O by placing 10 μL ddH_2O on the TEM grid and quickly wicking off on filter paper. After that, samples were negatively stained with 10 μL 2 wt% uranyl acetate for 20 seconds. The excessive uranyl acetate was wicked off and the grid was dried for 45 min under an infrared lamp (Zhongjingkeyi Technology). TEM images were obtained on a FEI T12 transmission electron microscope operated at 120 kV accelerating voltage.

Scanning electron microscopy (SEM) imaging

The industrial fillers were immersed in the bacteria cultivation solution and incubated at 29°C. After 2-3 days of cultivation, the fillers were taken out and washed three times with ddH_2O. For scanning electron microscopy (SEM), biofilm samples were sputtered with Pt at 30 mA for 45 seconds with a JEC-3000FC sputter coater. The samples were then imaged with a JSM-7800F Prime scanning electron microscope operated at 1.5 kV accelerating voltage.

Atomic force microscopy (AFM) imaging

The purified CsgA-C5 and CsgA solution was diluted to 1 mg/mL. A total of 150 μL of the solution was then incubated on mica surfaces for 18 h. The mica was rinsed with excess deionized water and dried using nitrogen flow. The sample was then imaged by AFM (Asylum MFP-3D) on AC air tapping mode using Veeco Probes Sb-doped Si cantilevers (ρ = 0.01-0.025 Ω-cm, k = 40 N/m, ν~ 300 kHz).

Biomass quantification of CsgA or CsgA-C5 nanofiber coatings formed on glass substrates.

We quantified the biomass of proteins coated on the substrate by measuring the initial

and remaining concentrations of protein solutions before and after nanofiber coating formation on the substrates. Specifically, concentration of the fresh-made CsgA or CsgA-C5 protein solution in TBS buffer (20 mM Tris, 800 mM imidazole, pH=7.4) was first measured via a Nanodrop 2000 Spectrophotometer (Thermo). Afterwards, 60 μL of the solution was dropped on clean glass slices (Φ=8 mm) and incubated in a petri dish overnight to allow sufficient formation of self-assembled fibers on the glass substrates. Several drops of ddH_2O were dripped in the petri dish to minimize the volatilization of the CsgA/CsgA-C5 protein solution. After nanofiber formation on the glass substrates, the remaining protein solution, along with a total of 120 μL TBS buffer used to wash the glass slides for 3 times, was collected into a centrifugation tube. The volume of the mixed solution was fixed at 180 μL and the protein concentration was measured via a Nanodrop 2000 Spectrophotometer (Thermo). The biomass amount for the two samples determined using this approach is 0.051 and 0.054 mg for the CsgA and CsgA-C5 sample, respectively. Therefore, we determine that 0.101 mg of CsgA and 0.107 mg of CsgA-C5 protein coatings form on 1 cm^2 glass surface (Supplementary able 1).

Enzyme linked immunosorbent assay (ELISA)

For the ELISA assay, we followed the general ELISA protocol of Sino Biological. For the interaction between HA protein and CsgA-C5 or CsgA proteins, 200 μL fresh purified CsgA-C5 or CsgA proteins (1 mg/mL) were incubated in a 24-well plate overnight at room temperature and then washed three times with 300 μL TBST (0.05% Tween 20 in TBS, pH=7.2). Then, the plate was blocked by adding 300 μL blocking buffer (2% BSA in TBST) in the well and incubated for 1 hour and washed three times with TBST. A total of 150 μL recombinant influenza A hemagglutinin (A/California/07/2009 purchased from Sino Biological, 2 ng/mL) in dilution buffer (0.5% BSA in TBST, pH=7.2, 0.2 μm filtered) was added to the plate to interact with CsgA-C5 or CsgA for 2 hours at room temperature and then the plate was washed three times with TBST. Detection antibody (150 μL, 0.8 μL/mL of rabbit

anti-influenza A H1N1 hemagglutinin/polyclonal antibody conjugated to horseradish-peroxidase) in dilution buffer was added to the plate and incubated for 2 hours, and then washed three times with 300 μL TBST. A total of 200 μL substrate solution (0.1 mg/mL TMB (tetramethylbenzidine), 0.05 M Na_2HPO4 and 0.025 M citric acid, 0.0024% hydrogen peroxide, pH=5.5) was added to the well and incubated for 7 min. H_2SO_4 (50 μL, 1 mol/L) was added to stop the reaction and the optical density at 450 nm was determined using a micro reader.

Cell culture and virus infection

Madin-Darby Canine Kidney (MDCK) cells were cultured in modified Eagle's medium (MEM) with 10% fetal bovine serum (FBS). MDCK cells ($1x10^5$) were seeded to the wall of a 6-well plate for over 12 hours. The MDCK cells were infected by influenza viruses (A/Puerto Rico/8/34 H1N1) then maintained in MEM with 0.3% bovine serum albumin (BSA) and 0.5 μg/ml tosylsulfonyl phenylalanyl chloromethyl ketone (TPCK)-trypsin. The purification and titer measurement of H1N1 PR8 viruses were carried out at Wuhan Institute of Virology following a published protocol[4]. Infectivity titers for all virus stocks were calculated as plaque forming unit (PFU) per mL.

Immunofluorescence imaging

1. For the immunofluorescence imaging of CsgA-C5 or CsgA protein fibers on glass slice binding hemagglutinin, 200 μL fresh purified CsgA-C5 or CsgA proteins (1 mg/mL) in Tris·HCl buffer (pH=7.2) were dripped on a glass slice and incubated overnight at room temperature. The protein coated glass was washed with TBS buffer (pH=7.2).

A total of 150 μL of recombinant influenza hemagglutinin (A/California/07/2009 purchased from Sino Biological, 2 ng/mL) in dilution buffer (0.5% BSA in TBST, pH=7.2, 0.2 μm filtered) was added to the glass to interact with CsgA-C5 or CsgA for 2 hours at room temperature and washed with TBS buffer (pH=7.2). Rabbit anti-influenza A H1N1 hemagglutinin/polyclonal antibody in TBS buffer (150 μL)

was dripped on the glass and incubated for 1 hour. After washing with TBS buffer, the glass was incubated with 150 μL Alexa 488-conjugated goat anti-rabbit secondary antibody for 1 h and washed with TBST. The prepared samples were observed with a Zeiss Z2 fluorescence microscope.

2. For the immunofluorescence imaging of CsgA-C5 and CsgA protein fibers (CsgA-C5 or CsgA biofilms) binding influenza virus particles, the prepared CsgA-C5 or CsgA protein fibers (biofilms) coating glass slices were incubated with 150 μL recombinant influenza hemagglutinin (A/California/07/2009 purchased from Sino Biological, 2 ng/mL) in dilution buffer (0.5% BSA in TBST, pH=7.2, 0.2 μm filtered) for 2 hours at room temperature and washed with TBS buffer (pH=7.2). Rabbit anti-influenza A H1N1 hemagglutinin (150 μL)/polyclonal antibody in TBS buffer was dripped on the glass and incubated for 1 hour. After washing with TBS buffer, the glass was incubated with 150 μL Alexa 488-conjugated goat anti-rabbit secondary antibody for 1 h and washed with TBST. The prepared samples were observed with a Zeiss Z2 fluorescence microscope.

3. For the immunofluorescence imaging of the influenza virus solution treated with or without CsgA-C5 biofilms infecting MDCK cells, $7x10^4$ PFU/mL influenza viruses (A/Puerto Rico/8/34 H1N1) in liquid M63 culture media were mixed with CsgA-C5 biofilm-forming *E. coli* cells (control experiments were influenza viruses in M63 media without *E. coli* cells) and co-cultured for 3 days at 29°C. The supernatant was taken out and filtered through a 220 nm filter, and 300 μL of the solution was added to infect the MDCK cells seeded in the 35 mm cell culture glass bottomed dishes. The samples were then washed three times with phosphate-buffered saline (PBS), and fixed with 4% formaldehyde, permeabilized with 0.1% Triton X-100 in PBS, and blocked with 10% FBS in PBS. The samples were incubated with mouse monoclonal antibody against nucleoprotein (NP) (1:500) (Abcam) at 37°C for 1 h and then with Alexa Fluor 647-labeled goat anti-mouse IgG (1:2000) (Cell Signaling Technology). The cell nuclei were conjugated by Hoechst33342 (1:1000) (Beyotime). The samples

were observed with an inverted fluorescence microscope (Olympus IX83).

Homology modelling and molecular docking

We used MODELLER[5] for homology modelling and Schrödinger to set up the initial fiber and docking model. The initial structure of CsgA was from the last frame of 1 μs molecular dynamics from a previous study[3]. The structure of HA protein was obtained from PDB structure (1HGG chain: C, D) with the deletion of original sialic acids. The initial structure of C5 peptide was gathered from the de novo peptide build in Schrodinger Suite. The highest scored docking result to HA protein sialic acid binding domain was chosen as a candidate of C5 peptide. We then built monomer and pentamer fibrillar CsgA-C5 based on the candidate by MODELLER. The best scored candidate was chosen from 50 generated structures of monomer and pentamer fibrillar CsgA-C5. Then we performed an induced fit docking (IFD) on monomer CsgA-C5 to HA protein to get the structure of complex. Docking results were performed by Glide XP[6] and IFD[7] modules in Schrodinger Suite.

Molecular dynamics simulation

We performed all atom molecular dynamics simulations (MD) for 3 systems. They are (1) CsgA-C5 monomer and HA complex (147579 atoms, 800ns), (2) CsgA-C5 monomer (36386 atoms, 3x1 μs), and (3) pentamer fibrillar CsgA-C5 (94680 atoms, 1 μs). The starting structures of system 1 was generated from the MODELLER as previous mentioned. The N terminal loop of CsgA-C5 was cut and capped the N termini with ACE residue. The starting structure of system 2 was the CsgA-C5 part from the result of system 1 with three parallel simulation for system 2. The starting structure of system 3 was built by MODELLER. AMBER99SB-ILDN force field was used during the MD. The systems were set up under a TIP3P water model by GROMACS[8] with a dodecahedron box (for monomer CsgA-C5 with a minimum water length of 15.0 Å surround the system) or triclinic box (for pentamer CsgA-C5 or interaction with HA with a minimum water length of 10.0 Å surround the system). The systems were set to neutral conditions with addition of Cl^- and Na^+ ions and to

finally 0.15 M NaCl. The energy minimization was reduced with a steepest descent method of 5000 steps, and 5 subsequent stages of equilibrium including 1 stage of constant volumn and temperature ensemble (NVT) simulation and 5 stages of constant pressure and temperature ensemble (NPT) simulation for different times (100 ps, 2 ns, 2 ns, 2 ns, 2 ns, 2 ns, respectively). The NVT used v-rescale thermostat (modified Berendsen thermostat) The NPT used Parrinello-Rahman pressure coupling method with isotropic pressure scaling for pressure control and v-rescale thermostat. Position restraint coefficient was set separately to heavy atoms on sidechain backbone during each state of NVT or NPT equilibrium (4000 kJ/mol·nm^2 on backbone and 2000 kJ/mol·nm^2 on sidechain during NVT, 2000 kJ/mol·nm^2 on backbone and 1000 kJ/mol·nm^2 on sidechain during NPT-1, 1000 kJ/mol·nm^2 on backbone and 500 kJ/mol·nm^2 on sidechain during NPT-2, 500 kJ/mol·nm^2 on backbone and 200 kJ/mol·nm^2 on sidechain during NPT-3, 200 kJ/mol·nm^2 on backbone and 50 kJ/mol·nm^2 on sidechain during NPT-4, 50 kJ/mol·nm^2 on backbone and 0 kJ/mol·nm^2 on sidechain during NPT-5). The 800 ns or 1 μs production simulation was performed without any position restraints at 303 K with time step of 2 fs. The cut-off of long-range nonbonding interactions was set at 10 Å. MM-GBSA[9] (Molecular Mechanics/Generalized Born Surface Area) score from Schrodinger Suite was used for analyzing MD results to get binding free energy of CsgA-C5 to HA protein during molecular dynamics. Visual Molecular Dynamics (VMD)[10] was used to capture 8 stable frame during 800ns MD of system (1) to calculate MM-GBSA binding energy (in kcal/mol). The N-terminal of the protein frame captured from VMD was modified with COO^- and then underwent a Protein Preparation Wizard with hydrogen bond network optimization and restrained minimization (RMSD 0.3 Å) in the Schrodinger Suite.

Hemagglutination inhibition assay

Virus at titers of 7×10^4 PFU/mL was seeded onto a 12 well plate in 3 mL M63 culture medium with and without addition of $Tc_{Receiver}$/CsgA-C5 and incubated at 29°C for 72 hours. A total of 100 μL supernatant from the culture medium was removed and

added to a V-bottom well then mixed with 100 μL 1% chicken red blood cells in PBS buffer. After incubation for 1 hour at 37°C, the plates were then photographed.

RNA extraction, reverse transcription, and qPCR

Total viral RNA was extracted from 200 μL virus solution with an EasyPure® Viral DNA/RNA kit (TransGen Biotech, Beijing, China Cat. # ER201-01) according to the manufacturer's protocol into 20 μL Nuclease-free water (NEB, Beijing, China Cat. #B1500L) and then reverse transcripted into cDNA with Takara® PrimeScript RT Master Mix (Perfect Real Time) (TaKaRa, Dalian, China Cat. # RR036A). According to the protocol provided by the manufacturer, a 10 μL system (2 μL 5× PrimeScript RT Master Mix, 500 ng total RNA, NEB Nuclease-free water to 10 μL) was incubated at 37°C for 15 minutes and then 85°C for 15 seconds. The cDNA was diluted to 100 μL with NEB Nuclease-free water for later experiments. The diluted cDNA and extra RNA was then stored immediately at -80°C.

q-PCR was performed using a Roche LightCycler® 96 system (Roche, Shanghai, China) employing a ChamQTM SYBR® qPCR Master Mix (Vazyme, Nanjing, China Cat. #Q411-02). The probe set of MCG_MP_F (5′-AGATGAGTCTTCTAACCGAGGTCG -3′)[11] and MCG_MP_R (5′-GCAAAGACACTTTCCAGTCCTG -3′) were used to amplify the Matrix protein segment of H1N1 into 100-bp products. The qPCR reaction system was set to a 10 μL total system (5 μL 2×ChamQ SYBR qPCR Master Mix, 0.2 μL each of forward and reverse primers, 1 μL Template cDNA, 3.6 μL NEB Nuclease-free water). qPCR response procedures (Stage 1: 1 cycle at 95°C for 30 s; Stage 2: 40 cycles at 95°C for 10 s, 60°C for 30 s; Stage 3: 1 cycle at 95°C for 15 s, 60°C for 60 s, 95°C for 15 s) were applied following the manufacturer's instructions.

Biomass quantification of CsgA-C5 biofilm coatings formed on industrial fillers.

The formation of CsgA-C5 biofilm coatings on industrial fillers was conducted by incubating ten pieces of fillers (Kaldnes K1, Φ=11 mm) in 100 mL M63 culture

media and then cultivated for 3 days at 29°C. The fillers were then taken out and washed with Tris·HCl buffer. In order to quantify the amount of CsgA-C5 biofilm coatings formed on the fillers, we immersed five pieces of fillers coated with or without CsgA-C5 biofilms into 5 mL Congo red solution (0.04 mg/mL) and incubated for 1 hour at room temperature. The mixed solution was added into 1.5 mL tubes and then centrifugated at 10,000 g for 5 minutes in an Eppendorf Centrifuge 4424. Concentration of Congo red in supernatant was quantified by light absorbance at 496 nm. The amount of Congo red bound by CsgA-C5 biofilms were quantified by subtracting the $A_{496\ nm}$ of biofilm-coated fillers from $A_{496\ nm}$ of bare fillers, and the value acquired is 0.146. According to Supplementary Figure 12, we determined that about 9.3 mg CsgA-C5 biofilms formed on these five industrial fillers (1.86 mg biofilms per filler).

Virus disinfection from river water using CsgA-C5 biofilm-coated industrial fillers.

Five pieces of CsgA-C5 biofilm-coated industrial fillers were put in a column tube and incubated with 10 mL influenza virus solution in river water at room temperature for at least 2 hours. The solution was then allowed to flow out at an average rate of 0.67 mL/s. The titers of virus solutions before and after treatment with biofilm-coated fillers were analyzed by qPCR assay.

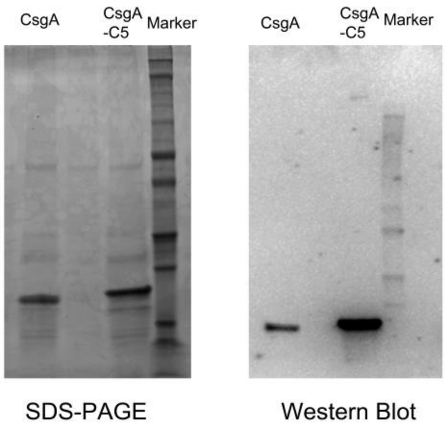

Supplementary Figure 1. Coomassie light blue stained SDS-PAGE gels and western blots with anti-His antibodies confirm purification of the expressed proteins by cobalt-resin columns.

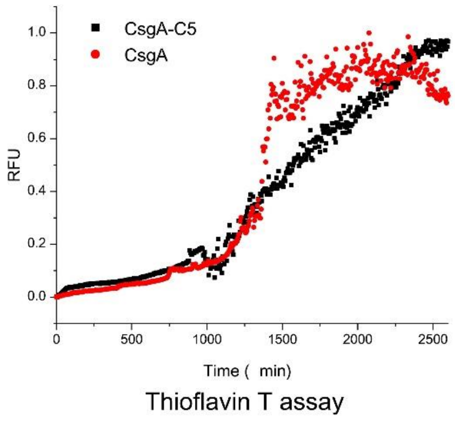

Supplementary Figure 2. ThT assay showing the assembly kinetics of amyloid CsgA and CsgA-C5 proteins over time.

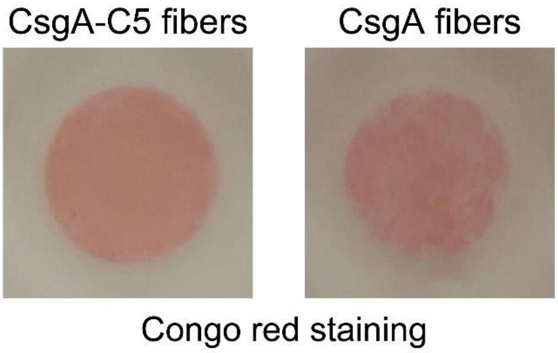

Supplementary Figure 3. Congo red staining showing the amyloid feature for CsgA-C5 and CsgA proteins.

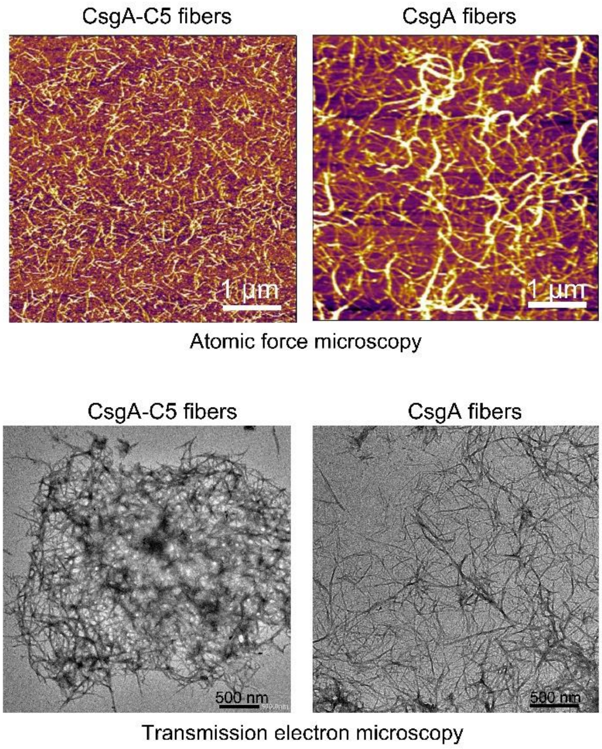

Supplementary Figure 4. AFM and TEM images showing morphologies of self-assembled CsgA and CsgA-C5 nanofibers. These data demonstrate that these two proteins can self-assemble into nanofibers.

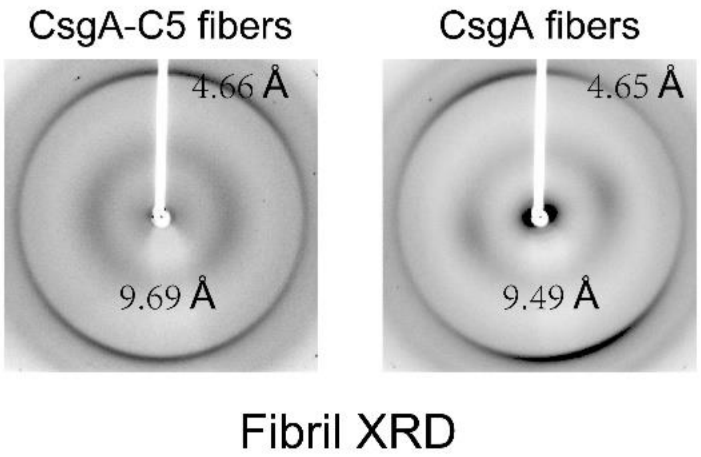

Supplementary Figure 5. Fibril X-ray diffraction (XRD) of CsgA and CsgA-C5 protein nanofibers. Both of these protein samples display typical cross-beta diffraction patterns, a pattern that is characteristic of amyloid fibrils[12]. Here, the meridional reflection at ~4.7 Å corresponds to the inter-β strand spacing and the equatorial reflection at ~6–11 Å corresponds to the distance between stacked β sheets[13].

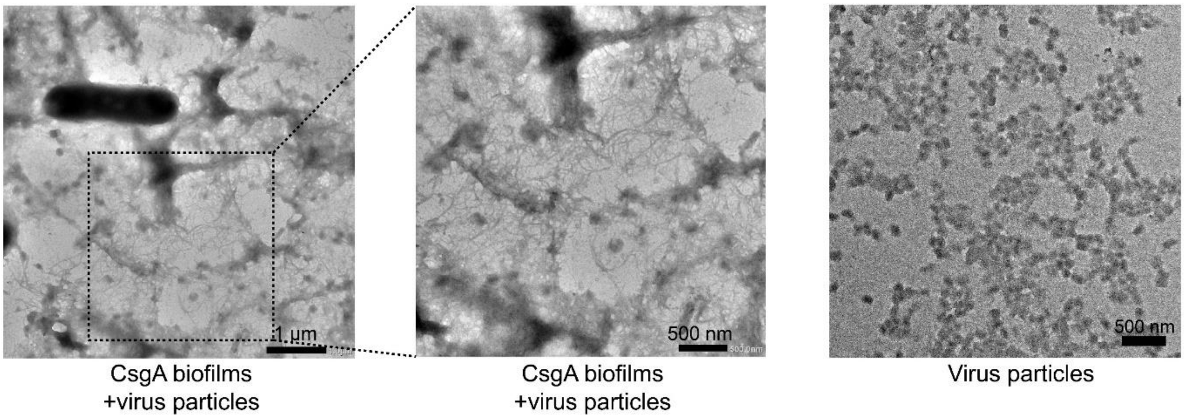

Supplementary Figure 6. TEM images showing CsgA biofilms binding influenza virus particles (A/Michigan/45/2015 H1N1) (left) and the influenza virus particles alone (right).

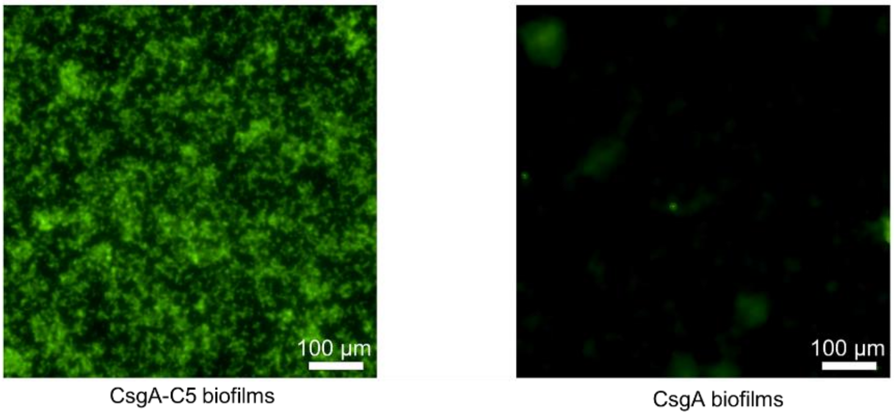

Supplementary Figure 7. Immunofluorescence images showing the different binding behaviors of influenza virus particles with CsgA-C5 and CsgA biofilms.

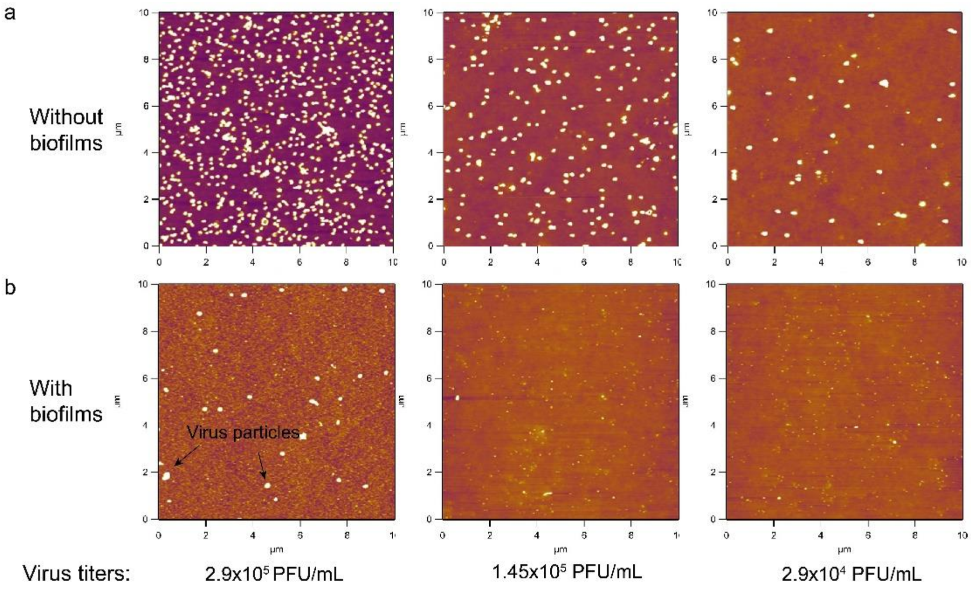

Supplementary Figure 8. AFM images of the supernatant of influenza virus solutions at gradient titers exposed to CsgA-C5 biofilms. Compared with the control groups, which are virus solutions without the addition of *E. coli* cells, the supernatant of the CsgA-C5 biofilms-treated virus solution contain obviously fewer virus particles.

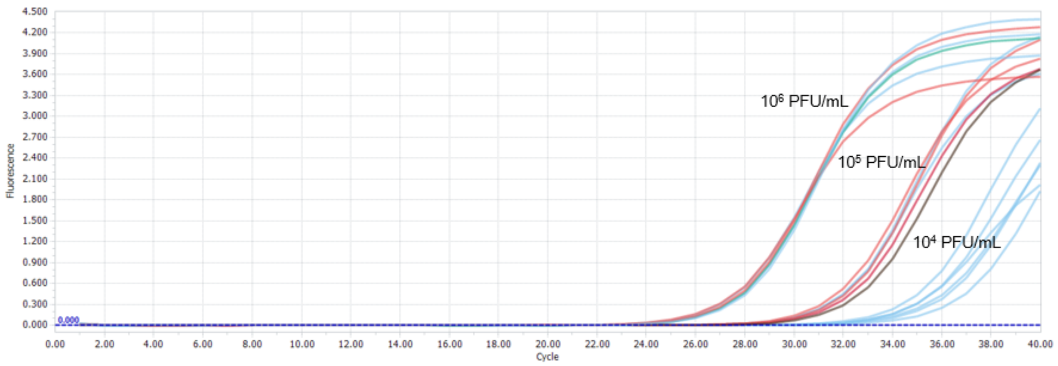

Supplementary Figure 9. The qPCR amplification curve of influenza viruses (A/Michigan/35/2015) at gradient titers of 10^6, 10^5, and 10^4 PFU/mL.

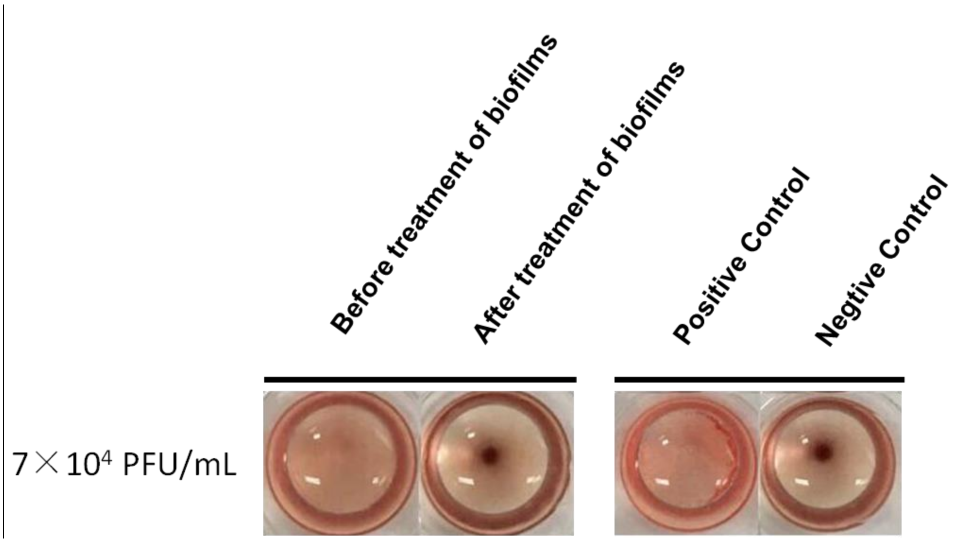

Supplementary Figure 10. Hemagglutination inhibition assay of influenza virus PR8 before and after CsgA-C5 biofilm treatment at titers of $7×10^4$ PFU/mL.

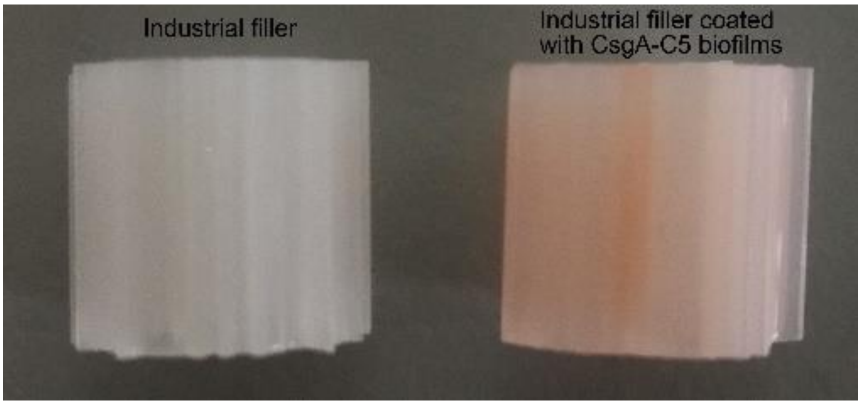

Supplementary Figure 11. Photograph of the Kaldnes K1 (Φ=11 mm) filler (an industrial filler on which bacteria can colonize) coated with the CsgA-C5 biofilms by immersing the filler in the M63 culture media inoculated with CsgA-C5 biofilm-forming *E. coli* cells for 3 days at 29°C. The fillers were taken out and washed with ddH_2O, then they were stained with 0.8 mg/mL Congo red for 10 min and washed with ddH_2O.

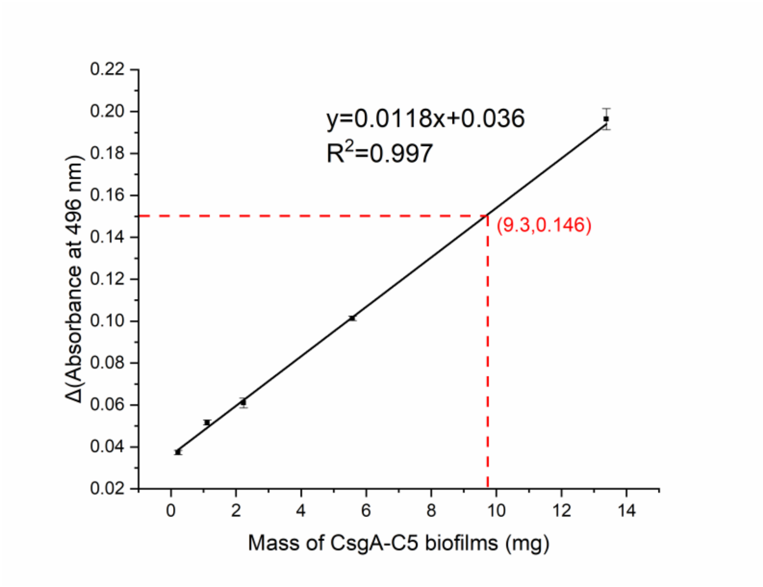

Supplementary Figure 12. A standard linear curve showing light absorption reduction of Congo red solution at 496 nm in proportion to the biomass of CsgA-C5 biofilms applied in the system. The red line represented the absorption reduction of Congo red solution and corresponding biomass amounts of CsgA-C5 biofilms formed on industrial fillers.

The standard curve representing the relationship between the Congo red absorbance change at 496 nm and the mass of CsgA-C5 biofilms was acquired through the following calibration experiment. Specifically, 0.223, 1.115, 2.23, 5,575 and 13.38 mg (wet weight) of CsgA-C5 biofilms were mixed with 5 mL Congo red solution (0.04 mg/mL) at room temperature for one hour and then the solution was centrifuged at 10,000 g for 5 minutes in an Eppendorf Centrifuge 4424. The amount of Congo red bound by CsgA-C5 biofilms was quantified by subtracting the $A_{496\ nm}$ of supernatant from $A_{496\ nm}$ of 0.4 mg/ml Congo red. The curve was plotted using a linear fit by OriginPro.

In order to quantify the biomass of CsgA-C5 biofilm coated on fillers, five pieces of fillers coated with or without CsgA-C5 biofilms were immersed into 5 mL Congo red solution (0.04 mg/mL) and incubated for 1 hour. The amount of Congo red bound by CsgA-C5 biofilms were quantified by subtracting the $A_{496\ nm}$ of biofilm-coated fillers from $A_{496\ nm}$ of the bare fillers. Here, we determined that about 9.3 mg CsgA-C5 biofilms formed on these five industrial fillers (1.86 mg biofilms per filler).

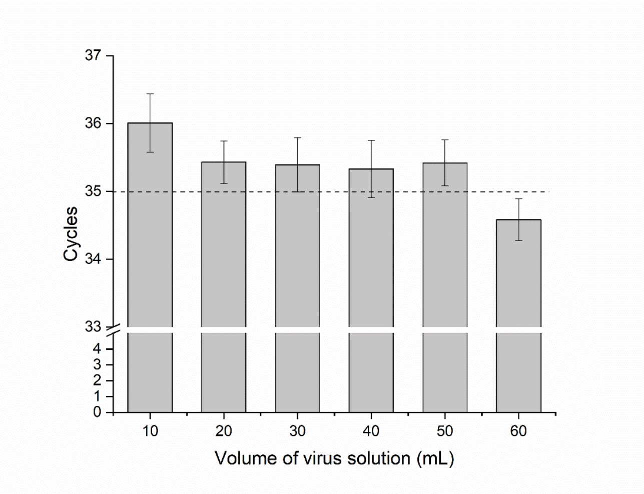

Supplementary Figure 13. qPCR analysis of the virus-spiked river water treated with CsgA-C5 biofilm-coated fillers. Results show means ± s.e.m. of three independent samples (n = 3). Five pieces of CsgA-C5 biofilm-coated fillers were incubated in the presence of excessive 10 mL influenza virus particles in river water (1.04×10^4 PFU/mL) at room temperature for at least 2 hours in a column tube. The solution was allowed to flow out at an average rate of 0.67 mL/s and the titers of virus solution were collected for analysis by qPCR assay.

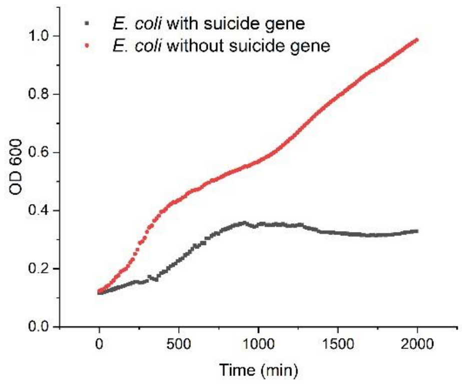

Supplementary figure 14. Growth curve of engineered bacteria with (black) or without (red) a suicide gene circuit. The engineered bacteria with or without a suicide gene circuit were inoculated into LB media at a volume ratio of 1:100 and cultured at 37°C. The cell density curve over time was monitored via a microplate reader (CYTATION, BioTek).

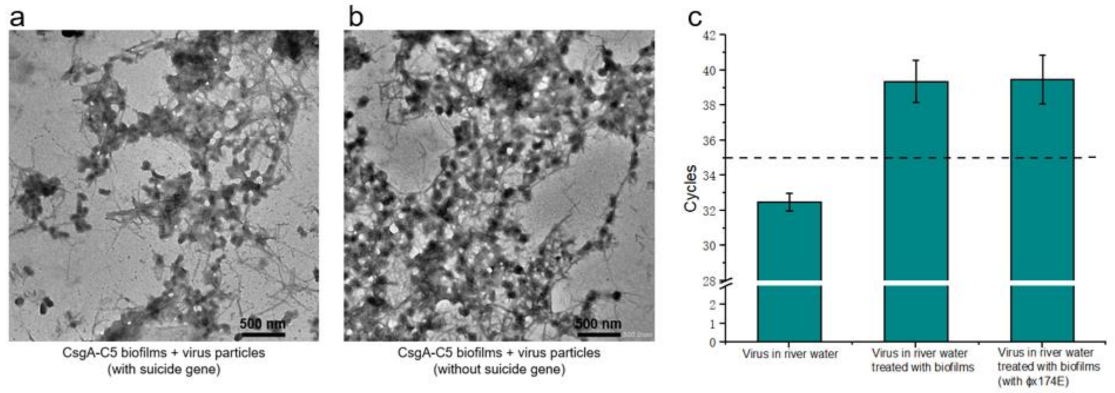

Supplementary figure 15. Virus disinfection from river water using engineered bacteria harboring a suicide gene circuit. TEM images showing binding of virus particles using the CsgA-C5 biofilms expressed by bacteria harboring a suicide gene circuit (a) or without a suicide gene circuit (b); c, qPCR analysis of the supernatants from virus samples (3.5×10^7 PFU/L) that were incubated with biofilms produced by bacteria with/without a suicide gene. Results show means ± s.e.m. of three independent samples (n = 3).

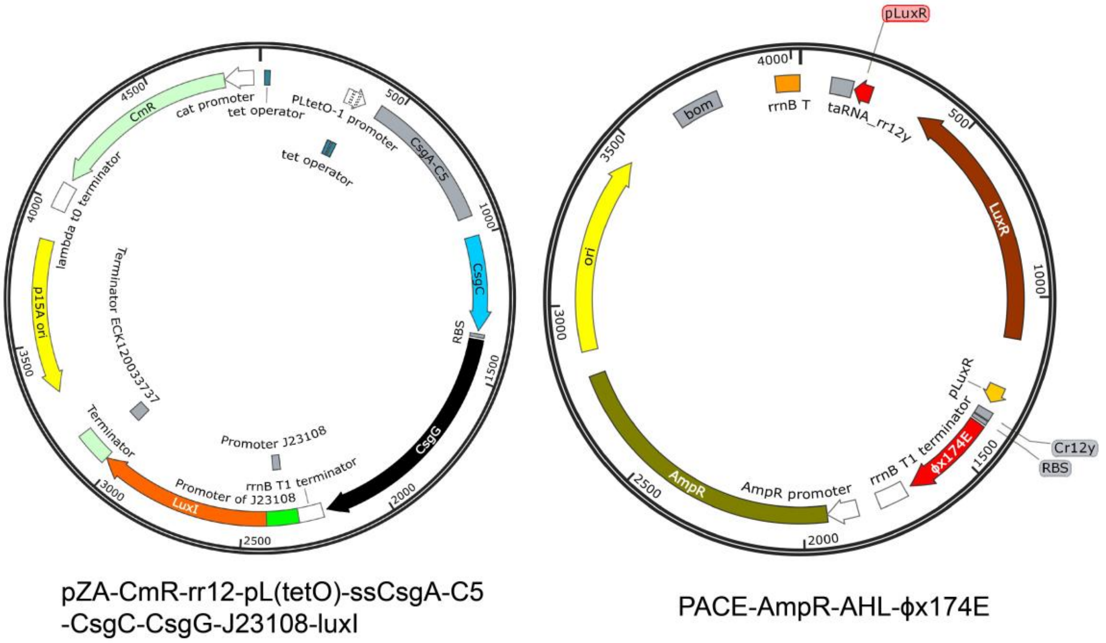

Supplementary figure 16. Plasmid maps of the constructed plasmids used to control the growth of the genetically engineered bacteria. These plasmids are constructed by assembling relative PCR fragments (listed in Supplementary Table 2) generated with Q5 polymerase via Gibson assembly.

Supplementary Table 1 | Biomass density for protein coatings formed on glass substrates, measured by concentration differences for CsgA-C5 or CsgA protein solutions before and after coating formation on glass substrates.

	CsgA-C5			CsgA		
Initial concentration (mg/mL)	1.376	1.398	1.381	1.289	1.323	1.354
Final concentration (mg/mL) (three times diluted)	0.167	0.163	0.161	0.154	0.159	0.162
Final concentration (mg/mL)	0.501	0.489	0.483	0.462	0.477	0.486
Biomass quantification of protein coatings formed on glass substrates (mg)	0.053	0.055	0.054	0.050	0.051	0.052
Biomass density of protein coatings formed on glass substrates (mg/cm^2)	0.107±0.0021			0.101±0.0025		

Supplementary Table 2 | Synthetic materials used in this work.

Name	Type	Sequence	Source
CsgA noSS	Gene encoding the mature form of CsgA protein without the secretion signal	ATGGGTGTTGTTCCTCAGT<br>ACGGCGGCGGCGGTAACC<br>ACGGTGGTGGCGGTAATA<br>ATAGCGGCCCAAAT<br>CACCATCACCATCACCACC<br>AT<br>TCTGAGCTGAACATTTACC<br>AGTACGGTGGCGGTAACTC<br>TGCACTTGCTCTGCAAACT<br>GATGCCCGTAACTCTGACT<br>TGACTATTACCCAGCATGG<br>CGGCGGTAATGGTGCAGAT<br>GTTGGTCAGGGCTCAGATG<br>ACAGCTCAATCGATCTGAC<br>CCAACGTGGCTTCGGTAAC<br>AGCGCTACTCTTGATCAGT<br>GGAACGGCAAAAATTCTG<br>AAATGAC<br>GGTTAAACAGTTCGGTGGT<br>GGCAACGGTGCTGCAGTTG<br>ACCAGACTGCATCTAACTC<br>CTCCGTCAACGTGACTCAG<br>GTTGGCTTTGGTAACAACG<br>CGACCGCTCATCAGTACTA<br>A	This work
CsgA-C5 noSS	Gene encoding the mature form of CsgA-C5 protein without the secretion signal	ATGGGTGTTGTTCCTCAGT<br>ACGGCGGCGGCGGTAACC<br>ACGGTGGTGGCGGTAATA<br>ATAGCGGCCCAAAT<br>CACCATCACCATCACCACC<br>AT<br>TCTGAGCTGAACATTTACC<br>AGTACGGTGGCGGTAACTC<br>TGCACTTGCTCTGCAAACT<br>GATGCCCGTAACTCTGACT<br>TGACTATTACCCAGCATGG<br>CGGCGGTAATGGTGCAGAT<br>GTTGGTCAGGGCTCAGATG<br>ACAGCTCAATCGATCTGAC<br>CCAACGTGGCTTCGGTAAC<br>AGCGCTACTCTTGATCAGT<br>GGAACGGCAAAAATTCTG	This work

		AAATGAC<br>GGTTAAACAGTTCGGTGGT<br>GGCAACGGTGCTGCAGTTG<br>ACCAGACTGCATCTAACTC<br>CTCCGTCAACGTGACTCAG<br>GTTGGCTTTGGTAACAACG<br>CGACCGCTCATCAGTACGC<br>GCGTCTGCCGCGTTAA	
CsgA	Gene encoding the mature form of CsgA protein fused with the secretion signal	ATGAAACTTTTAAAAGTAG<br>CAGCAATTGCAGCAATCGT<br>ATTCTCCGGTAGCGCTCTG<br>GCA<br>GGTGTTGTTCCTCAGTACG<br>GCGGCGGCGGTAACCACG<br>GTGGTGGCGGTAATAATAG<br>CGGCCCAAAT<br>CACCATCACCATCACCACC<br>AT<br>TCTGAGCTGAACATTTACC<br>AGTACGGTGGCGGTAACTC<br>TGCACTTGCTCTGCAAACT<br>GATGCCCGTAACTCTGACT<br>TGACTATTACCCAGCATGG<br>CGGCGGTAATGGTGCAGAT<br>GTTGGTCAGGGCTCAGATG<br>ACAGCTCAATCGATCTGAC<br>CCAACGTGGCTTCGGTAAC<br>AGCGCTACTCTTGATCAGT<br>GGAACGGCAAAAATTCTG<br>AAATGAC<br>GGTTAAACAGTTCGGTGGT<br>GGCAACGGTGCTGCAGTTG<br>ACCAGACTGCATCTAACTC<br>CTCCGTCAACGTGACTCAG<br>GTTGGCTTTGGTAACAACG<br>CGACCGCTCATCAGTACTA<br>A	This work
CsgA-C5	Gene encoding the mature form of CsgA-C5 protein fused with the secretion signal	ATGAAACTTTTAAAAGTAG<br>CAGCAATTGCAGCAATCGT<br>ATTCTCCGGTAGCGCTCTG<br>GCA<br>GGTGTTGTTCCTCAGTACG<br>GCGGCGGCGGTAACCACG<br>GTGGTGGCGGTAATAATAG	This work

		CGGCCCAAAT CACCATCACCATCACCACC AT TCTGAGCTGAACATTTACC AGTACGGTGGCGGTAACTC TGCACTTGCTCTGCAAACT GATGCCCGTAACTCTGACT TGACTATTACCCAGCATGG CGGCGGTAATGGTGCAGAT GTTGGTCAGGGCTCAGATG ACAGCTCAATCGATCTGAC CCAACGTGGCTTCGGTAAC AGCGCTACTCTTGATCAGT GGAACGGCAAAAATTCTG AAATGAC GGTTAAACAGTTCGGTGGT GGCAACGGTGCTGCAGTTG ACCAGACTGCATCTAACTC CTCCGTCAACGTGACTCAG GTTGGCTTTGGTAACAACG CGACCGCTCATCAGTACGC GCGTCTGCCGCGTTAA	
CsgC	Gene encoding the CsgC protein, which can facilitate the secretion of CsgA-C5 in M63 culture media	ATGAATACGTTATTACTCC TTGCGGCACTTTCCAGTCA GATAACCTTTAATACGACC CAGCAAGGGGATGTGTAT ACCATTATTCCTGAAGTCA CTCTTACTCAATCTTGTCTG TGCAGAGTACAAATATTGT CCCTGCGCGAAGGCAGTTC AGGGCAAAGTCAGACGAA GCAAGAAAAGACCCTTTCA TTGCCTGCTAATCAACCCA TTGCTTTGACGAAGTTGAG TTTAAATATTTCCCCGGAC GATCGGGTGAAAATAGTTG TTACTGTTTCTGATGGACA GTCACTTCATTTATCACAA CAATGGCCGCCCTCTTCAG AAAAGTCTTAA	[14]
CsgG	Gene encoding the CsgG protein, which can facilitate the secretion of CsgA-C5 in M63 culture media	ATGCAGCGCTTATTTCTTTT GGTTGCCGTCATGTTACTG AGCGGATGCTTAACCGCCC CGCCTAAAGAAGCCGCCA	[14]

		GACCGACATTAATGCCTCG<br>TGCTCAGAGCTACAAAGAT<br>TTGACCCATCTGCCAGCGC<br>CGACGGGTAAAATCTTTGT<br>TTCGGTATACAACATTCAG<br>GACGAAACCGGGCAATTT<br>AAACCCTACCCGGCAAGTA<br>ACTTCTCCACTGCTGTTCC<br>GCAAAGCGCCACGGCAAT<br>GCTGGTCACGGCACTGAAA<br>GATTCTCGCTGGTTTATAC<br>CGCTGGAGCGCCAGGGCTT<br>ACAAAACCTGCTTAACGAG<br>CGCAAGATTATTCGTGCGG<br>CACAAGAAAACGGCACGG<br>TTGCCATTAATAACCGAAT<br>CCCGCTGCAATCTTTAACG<br>GCGGCAAATATCATGGTTG<br>AAGGTTCGATTATCGGTTA<br>TGAAAGCAACGTCAAATCT<br>GGCGGGGTTGGGGCAAGA<br>TATTTTGGCATCGGTGCCG<br>ACACGCAATACCAGCTCGA<br>TCAGATTGCCGTGAACCTG<br>CGCGTCGTCAATGTGAGTA<br>CCGGCGAGATCCTTTCTTC<br>GGTGAACACCAGTAAGAC<br>GATACTTTCCTATGAAGTT<br>CAGGCCGGGGTTTTCCGCT<br>TTATTGACTACCAGCGCTT<br>GCTTGAAGGGGAAGTGGG<br>TTACACCTCGAACGAACCT<br>GTTATGCTGTGCCTGATGT<br>CGGCTATCGAAACAGGGG<br>TCATTTTCCTGATTAATGA<br>TGGTATCGACCGTGGTCTG<br>TGGGATTTGCAAAATAAAG<br>CAGAACGGCAGAATGACA<br>TTCTGGTGAAATACCGCCA<br>TATGTCGGTTCCACCGGAA<br>TCCTGA	
cmR	cmR	ATGGAGAAAAAAATCACT<br>GGATATACCACCGTTGATA<br>TATCCCAATGGCATCGTAA	[15]

		AGAACATTTTGAGGCATTT CAGTCAGTTGCTCAATGTA CCTATAACCAGACCGTTCA GCTGGATATTACGGCCTTT TTAAAGACCGTAAAGAAA AATAAGCACAAGTTTTATC CGGCCTTTATTCACATTCTT GCCCGCCTGATGAATGCTC ATCCGGAATTCCGTATGGC AATGAAAGACGGTGAGCT GGTGATATGGGATAGTGTT CACCCTTGTTACACCGTTT TCCATGAGCAAACTGAAAC GTTTTCATCGCTCTGGAGT GAATACCACGACGATTTCC GGCAGTTTCTACACATATA TTCGCAAGATGTGGCGTGT TACGGTGAAAACCTGGCCT ATTTCCCTAAAGGGTTTAT TGAGAATATGTTTTTCGTC TCAGCCAATCCCTGGGTGA GTTTCACCAGTTTTGATTT AAACGTGGCCAATATGGA CAACTTCTTCGCCCCCGTT TTCACCATGGGCAAATATT ATACGCAAGGCGACAAGG TGCTGATGCCGCTGGCGAT TCAGGTTCATCATGCCGTC TGTGATGGCTTCCATGTCG GCAGAATGCTTAATGAATT ACAACAGTACTGCGATGA GTGGCAGGGCGGGGCGTA A	
pL(tetO)	Promoter	TCCCTATCAGTGATAGAGA TTGACATCCCTATCAGTGA TAGAGATACTGAGCACATC AGCAGGACGCACTGACC	[15]
rrnB T1	Terminator	GGCATCAAATAAAACGAA AGGCTCAGTCGAAAGACT GGGCCTTTCGTTTTATCTGT TGTTTGTCGGTGAACGCTC TCCTGAGTAGGACAAATCC GCCGCCCTAGA	[16]
J2301	Promoter	GCTTGCATGCCTGCAGGTC	[17]

8		GACTCTAGAGGATCCCCGG GTACCCTGACAGCTAGCTC AGTCCTAGGTATAATGCTA GCTCTACCCGTCAATCAAT CATTTAGGAAGGTTAGCA	
ECK1 20033 737	Terminator	TAATAATCTAGAGGAAAC ACAGAAAAAAGCCCGCAC CTGACAGTGCGGGCTTTTT TTTTCGACCAAAGGCTGTA GAACGGAGTAACCCGGTG TGCGGTTGTATGCCTGCTG TGGATTG	
luxI	LuxI	ATGACTATAATGATAAAAA AATCGGATTTTTTGGCAAT TCCATCGGAGGAGTATAAA GGTATTCTAAGTCTTCGTT ATCAAGTGTTTAAGCAAAG ACTTGAGTGGGACTTAGTT GTAGAAAATAACCTTGAAT CAGATGAGTATGATAACTC AAATGCAGAATATATTTAT GCTTGTGATGATACTGAAA ATGTAAGTGGATGCTGGCG TTTATTACCTACAACAGGT GATTATATGCTGAAAAGTG TTTTTCCTGAATTGCTTGGT CAACAGAGTGCTCCCAAA GATCCTAATATAGTCGAAT TAAGTCGTTTTGCTGTAGG TAAAAATAGCTCAAAGAT AAATAACTCTGCTAGTGAA ATTACAATGAAACTATTTG AAGCTATATATAAACACGC TGTTAGTCAAGGTATTACA GAATATGTAACAGTAACAT CAACAGCAATAGAGCGAT TTTTAAAGCGTATTAAAGT TCCTTGTCATCGTATTGGA GACAAAGAAATTCATGTAT TAGGTGACACTAAATCGGT TGTATTGTCTATGCCTATT AATGAACAGTTTAAAAAA GCAGTCTTAAATGCTGCAA ACGACGAAAACTACGCTTT	[18]

		AGTAGCTTAA	
luxR	LuxR	ATGAAAAACATAAATGCC GACGACACATACAGAATA ATTAATAAAATTAAAGCTT GTAGAAGCAATAATGATAT TAATCAATGCTTATCTGAT ATGACTAAAATGGTACATT GTGAATATTATTTACTCGC GATCATTTATCCTCATTCT ATGGTTAAATCTGATATTT CAATTCTAGATAATTACCC TAAAAAATGGAGGCAATA TTATGATGACGCTAATTTA ATAAAATATGATCCTATAG TAGATTATTCTAACTCCAA TCATTCACCAATTAATTGG AATATATTTGAAAACAATG CTGTAAATAAAAAATCTCC AAATGTAATTAAAGAAGC GAAAACATCAGGTCTTATC ACTGGGTTTAGTTTCCCTA TTCATACGGCTAACAATGG CTTCGGAATGCTTAGTTTT GCACATTCAGAAAAAGAC AACTATATAGATAGTTTAT TTTTACATGCGTGTATGAA CATACCATTAATTGTTCCT TCTCTAGTTGATAATTATC GAAAAATAAATATAGCAA ATAATAAATCAAACAACG ATTTAACCAAAAGAGAAA AAGAATGTTTAGCGTGGGC ATGCGAAGGAAAAAGCTC TTGGGATATTTCAAAAATA TTAGGCTGCAGTGAGCGTA CTGTCACTTTCCATTTAAC CAATGCGCAAATGAAACTC AATACAACAAACCGCTGCC AAAGTATTTCTAAAGCAAT TTTAACAGGAGCAATTGAT TGCCCATACTTTAAAAATT AA	[18]
ϕx174 E	Lysis protein	ATGGTACGCTGGACTTTGT GGGATACCCTCGCTTTCCT	[18]

		GCTCCTGTTGAGTTTATTG<br>CTGCCGTCATTGCTTATTA<br>TGTTCATCCCGTCAACATT<br>CAAACGGCCTGTCTCATCA<br>TGGAAGGCGCTGAATTTAC<br>GGAAAACATTATTAATGGC<br>GTCGAGCGTCCGGTTAAAG<br>CCGCTGAATTGTTCGCGTT<br>TACCTTGCGTGTACGCGCA<br>GGAAACACTGACGTTCTTA<br>CTGACGCAGAAGAAAACG<br>TGCGTCAAAAATTACGTGC<br>GGAAGGAGTGA	

Supplementary Table 3 | Plasmids used in this work.

Plasmid name	Description	Source
pZA-CmR-rr12-pL(tetO)-CsgA	p15A origin, Cm resistance, rr12 riboregulator, pL(tetO) promoter, CsgA output gene	This work
pZA-CmR-rr12-pL(tetO)-CsgA-C5-CsgC-CsgG	p15A origin, Cm resistance, rr12 riboregulator, pL(tetO) promoter, CsgA-C5, CsgC and CsgG output genes	This work
pET-22b-CsgA noSS	CsgA without secretion signal inserted in the expressing region of the commercially available pET-22b vector (Novagen)	This work
pET-22b-CsgA-C5 noSS	CsgA-C5 without secretion signal inserted in the expressing region of the commercially available pET-22b vector (Novagen)	This work
pZA-CmR-rr12-pL(tetO)-ssCsgA-C5 -CsgC-CsgG-J23108-luxI	p15A origin, Cm resistance, rr12 riboregulator, pL(tetO) promoter, CsgA-C5, CsgC and CsgG output genes and J23018 promoter, LuxI output gene	This work
PACE-AmpR-AHL-ϕx174E	pBR322 origin, Amp resistnce, LuxR regulator, pLuxR promoter, ϕx174E output gene	This work

Supplementary Table 4 | Strains used in this work.

Strain name	Description	Antibiotic resistance	Source
MG1655 PRO ΔCsgA ompR234	*E. coli* strain with constitutive high level expression of tetR and lacI from PRO cassette derived from pZS4Int-lacI/tetR, with CsgA knocked out, and with ompR234 mutation that confers ability to produce curli fibrils in liquid M63 minimal media.	Spec, Kan	[1]
$Tc_{Receiver}$/CsgA	*E. coli* strain that expresses CsgA under tight regulation by a tetracycline (Tc) inducer-responsive riboregulator. Made by transforming pZA-CmR-rr12-pL(tetO)-CsgA plasmid into MG1655 PRO ΔCsgA ompR234.	Spec, Kan, Cm	This work
$Tc_{Receiver}$/CsgA-C5	*E. coli* strain that expresses CsgA-C5 under tight regulation by a tetracycline (Tc) inducer-responsive riboregulator. Made by transforming pZA-CmR-rr12-pL(tetO)-CsgA-C5-CsgC-CsgG plasmid into MG1655 PRO ΔCsgA ompR234.	Spec, Kan, Cm	This work
$Tc_{Receiver}$/CsgA-C5 /suicide	*E. coli* strain that expresses CsgA-C5 under tight regulation by a tetracycline (Tc) inducer-responsive riboregulatory and suicides when the cell density reaches a critical threshold. Made by transforming pZA-CmR-rr12-pL(tetO)-CsgA-C5-CsgC-CsgG plasmid and PACE-AmpR-AHL-ϕx174E into MG1655 PRO ΔCsgA ompR234.	Spec, Kan, Cm, Amp	This work
$IPTG_{Reciver}$/CsgA	Commercially available BL21(DE3) that expresses CsgA (without secretion signal) under tight regulation by isopropyl β-D-thiogalactoside (IPTG) inducer. Made by transforming pET-22b-CsgA plasmid into BL21(DE3) commercial strain.	Amp	This work
$IPTG_{Reciver}$/CsgA-C5	Commercially available BL21(DE3) that expresses CsgA-C5 (without secretion signal) under tight regulation by isopropyl β-D-thiogalactoside (IPTG) inducer. Made by transforming pET-22b-CsgA-C5 plasmid	Amp	This work

	into BL21(DE3) commercial strain.		

Supplementary Table 5 | Primers used to detect influenza viruses in solution via qPCR

Name	Sequence	Source
Influenza virus MP-F	AGATGAGTCTTCTAACCGAGGTCG	[11]
Influenza virus MP-R	GCAAAGACACTTTCCAGTCTCTG	This work

References:

[1] A. Y. Chen, Z. Deng, A. N. Billings, U. O. Seker, M. Y. Lu, R. J. Citorik, B. Zakeri, T. K. Lu, *Nat Mater* **2014**, *13*, 515.

[2] K. A. Datsenko, B. L. Wanner, *Proc Natl Acad Sci U S A* **2000**, *97*, 6640.

[3] C. Zhong, T. Gurry, A. A. Cheng, J. Downey, Z. Deng, C. M. Stultz, T. K. Lu, *Nat Nanotechnol* **2014**, *9*, 858.

[4] C. Qin, W. Li, Q. Li, W. Yin, X. Zhang, Z. Zhang, X. E. Zhang, Z. Cui, *Proc Natl Acad Sci U S A* **2019**, *116*, 2577.

[5] A. Sali, *Molecular Medicine Today* **1995**.

[6] M. P. Repasky, M. Shelley, R. A. Friesner, *Current Protocols in Bioinformatics* **2007**, *18*, 8.12. 1.

[7] T. D. Woody Sherman, Matthew P. Jacobson, Richard A. Friesner, and Ramy Farid, *Journal of Medicinal Chemistry* **2006**, *49*.

[8] M. J. Abraham, T. Murtola, R. Schulz, S. Páll, J. C. Smith, B. Hess, E. Lindahl, *SoftwareX* **2015**, *1-2*, 19.

[9] N. Huang, C. Kalyanaraman, K. Bernacki, M. P. Jacobson, *Physical Chemistry Chemical Physics* **2006**, *8*, 5166.

[10] D. A. Humphrey W, Schulten K., *Journal of molecular graphics* **1996**, *14*, 33.

[11] A. Das, E. Spackman, D. Senne, J. Pedersen, D. L. Suarez, *J Clin Microbiol* **2006**, *44*, 3065.

[12] J. Greenwald, R. Riek, *Structure* **2010**, *18*, 1244.

[13] M. Sunde, L. C. Serpell, M. Bartlam, P. E. Fraser, M. B. Pepys, C. C. Blake, *Journal of molecular biology* **1997**, *273*, 729.

[14] N.-M. Dorval Courchesne, A. Duraj-Thatte, P. K. R. Tay, P. Q. Nguyen, N. S. Joshi, *ACS Biomaterials Science & Engineering* **2016**, *3*, 733.

[15] R. L. a. H. Bujard, *Nucleic Acids Research* **1997**, *25*.

[16] C. R. C. Jarred M. Callura, and James J. Collins, *Proc Natl Acad Sci U S A* **2012**, *109*.

[17] Q. Yan, S. S. J. J. o. b. e. Fong, **2017**, *11*, 33.

[18] S. Chowdhury, S. Castro, C. Coker, T. E. Hinchliffe, N. Arpaia, T. Danino, *Nat Med* **2019**, *25*, 1057.
